# Astrocytic TIA1‐Mediated Stress Granules Promote the Demyelination of Optic Neuritis by Sequestering mRNA of Cholesterol Synthesis Genes in an Experimental Autoimmune Encephalomyelitis Model

**DOI:** 10.1002/advs.202520299

**Published:** 2026-02-27

**Authors:** Zheyu Fang, Xin Hua, Ziwei Fan, Jiaxin Zhao, Yuan Zhang, Qiaoyi Zhang, Xi Qu, Shengli Pan, Bingbing Wan, Shiyin Yang, Jia Li, Xu Zhang

**Affiliations:** ^1^ Department of Neurology The First Affiliated Hospital of Wenzhou Medical University Wenzhou Zhejiang China; ^2^ Department of Neurology The Second Affiliated Hospital and Yuying Children's Hospital of Wenzhou Medical University Wenzhou Zhejiang China; ^3^ Department of Neurology Xuanwu Hospital Capital Medical University National Center for Neurological Disorders Beijing China; ^4^ Department of Orthopedics (Spine Surgery) The First Affiliated Hospital of Wenzhou Medical University Wenzhou Zhejiang China; ^5^ Department of Hepatobiliary and Pancreatic Surgery Department of Surgery School of Medicine Fourth Affiliated Hospital Zhejiang University Yiwu Zhejiang China

**Keywords:** cholesterol synthesis, demyelination, experimental autoimmune encephalomyelitis, stress granules, T‐cell intracellular antigen 1

## Abstract

Optic neuritis (ON) is a common manifestation of multiple sclerosis (MS), characterized by inflammation and demyelination of the optic nerves. Recent studies indicate increased expression of genes associated with stress granules (SGs) in MS, with T cell intracellular antigen 1 (TIA1) being a key component of SGs. However, the role of TIA1‐mediated SGs in MS‐ON remains unclear. In experimental autoimmune encephalomyelitis (EAE) mice, a model of MS, TIA1 and G3BP1^+^ (a cell marker of SGs) SGs were upregulated in retinal neurons and optic nerve astrocytes. Knockout of *Tia1* in the CNS (*Tia1* ^Nestin^‐CKO mice) suppressed demyelination, inflammatory infiltration, and retinal ganglion cells (RGCs) loss in EAE mice. Mechanically, RNA‐sequencing analysis revealed upregulation of cholesterol synthesis genes such as *hmgcs1* in *Tia1* ^Nestin^‐CKO EAE mice. Furthermore, deletion of *Tia1* in astrocytes (*Tia1* ^GFAP^‐CKO mice) also alleviated demyelination in optic nerves in EAE mice through decreasing SG formation and increasing HMGCS1 expression in Tia1^−/−^ astrocytes by decreasing the sequestration of *hmgcs1* mRNA into SGs. Treatment with diarylpropionitrile (DPN), an ERβ‐ligand, partially restored demyelination in *Tia1* ^GFAP^‐CKO EAE mice. These findings uncover the role of TIA1‐mediated SG dynamics in MS‐ON, highlighting a novel mechanism and potential therapeutic target for treatment.

## Graphical abstract

Astrocytic TIA1‐mediated stress granules (SGs) promote demyelination by sequestering the mRNA of cholesterol synthesis genes. In this model, astrocytic TIA1‐mediated SGs were increased in the optic nerves of EAE mice, leading to the downregulation of cholesterol synthesis genes such as *HMGCS1* through sequestration of their mRNA into SGs, which exacerbated the demyelination of the optic nerves in EAE mice.

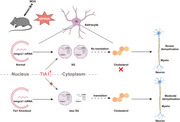

## 1 Introduction

Multiple sclerosis (MS) is a chronic inflammatory demyelinating disorder of the central nervous system (CNS) [1], characterized by diverse clinical phenotypes, among which relapsing‐remitting MS is the most prevalent. Optic neuritis (ON) is one of the main symptoms of MS, with approximately 1 out of 4 MS patients experiencing it as their initial symptom [2], and around 70% of cases experiencing it during the course of the disease, predominantly during relapses [3]. It presents as subacute visual impairment, retro‐orbital pain exacerbated by eye movement, central scotoma, reduced contrast sensitivity, accompanied by visual acuity decline, monocular visual loss, visual field defects, visual defects, afferent pupillary defects, and even complete visual loss [4]. Currently, effective treatments for permanent visual loss or damage caused by ON in MS are lacking [5].

MS‐ON is believed to be immune‐mediated, resulting in demyelination and damage to both myelin sheaths and nerve fibers. It initially presents as inflammation and demyelination of the optic nerves, followed by degeneration of the optic nerves and retinal ganglion cells [6, 7, 8]. Recent studies have shown that stress granules (SGs) play a crucial role in the pathogenesis of neurodegenerative diseases [9, 10, 11], with a close association between neuronal death and the accumulation of SGs in the cytoplasm [12]. Molecular analysis indicates an increased expression of SGs‐related genes in MS patients, and immunoreactive large SGs formation in neurons from MS brains, suggesting a potential basis for neuronal damage in MS. Additionally, SGs formation has been detected in astrocytes within the lesions, further implicating SGs in the MS pathology [13, 14]. In experimental autoimmune encephalomyelitis (EAE) models, there is an increased formation of SGs in neurons, astrocytes, oligodendrocytes, and microglia within and around demyelinating plaques [15]. However, the detailed functions and mechanisms of SGs in MS‐ON remain unknown.

SGs are membraneless cytoplasmic RNA granules containing translationally stalled mRNAs, associated translation initiation factors, and multiple RNA‐binding protein species (RBPs) [10, 11]. Upon exposure to stress, the translation initiation factor eIF2α undergoes phosphorylation, leading to selective sequestration of most cellular mRNAs into SGs while excluding other mRNAs required for the stress response [10, 16, 17]. SGs reversibly sequester translationally stalled mRNAs to maintain transcriptome homeostasis, minimizing energy expenditure and diverting metabolic resources to repair stress‐induced damage [11, 18]. The assembly of SGs is a multi‐step process involving the participation of SGs nucleating proteins such as T‐cell intracellular antigen‐1 (TIA1), Ras GTPase‐activating protein‐binding protein 1 (G3BP1), and TIA1‐related protein (TIAR) [19]. When the stress period ceases, SGs are disassembled, and the stored mRNAs can either return to polyribosomes for retranslation or be targeted for degradation in processing bodies [10, 18, 19].

In recent years, driven by advances in high‐throughput sequencing technologies and their application in large‐scale patient‐control cohort studies, RNA‐binding protein TIA1 with low‐complexity domains has been recognized as a key component of SGs and a major pathogenic gene in various neurological disorders [20]. TIA1 is a functional prion‐like protein containing a C‐terminal prion‐related domain essential for driving protein aggregation and SGs assembly, along with an N‐terminal RNA recognition motif that binds target mRNAs and recruits them to SGs [18, 21]. Therefore, the TIA1 protein is also considered a marker for SG stability. Under normal conditions, TIA1 protein shuttles dynamically between the cell nucleus and cytoplasm, but during stress, it accumulates in the cytoplasm [17, 18]. Interestingly, recent studies have found that in the brains of MS patients, TIA1 is translocated from neuronal cell nuclei, aggregates in the cytoplasm, and forms SGs [14]. However, the roles and mechanisms of TIA1‐mediated SGs in MS‐ON remain unknown.

In this study, we found that TIA1 was upregulated in retinal neurons and optic nerve astrocytes of EAE mice. TIA1 deletion in the CNS ameliorated the demyelination and neuroinflammation, reduced activation of astrocytes and microglia both in the optic nerves and retinas of EAE mice, accompanied by a decrease of loss of RGCs in EAE mice. Mechanistically, TIA1‐mediated SGs were upregulated in astrocytes of EAE mice and sequestered the mRNA of cholesterol synthesis genes such as *HMGCS1* into SGs, which aggravated the demyelination of the optic nerves in EAE mice. Our study reveals a potential mechanism whereby astrocytic TIA1‐mediated SGs promote the demyelination and neuroinflammation in MS‐ON through sequestration of cholesterol synthesis genes mRNA, which may contribute to the development of novel strategies for treating MS‐ON.

## 2 Materials and Methods

### 2.1 Animals Breeding and Genotyping

*Tia1* ^Nestin^‐CKO mice were generated by crossing the floxed *Tia1* allele (*Tia1* ^f/f^) mice (Shanghai Biomodel Organism Science & Technology Development Co., Ltd.) with Nestin‐Cre transgenic mice (Jackson lab), which expressed Cre recombinase in neural stem cells under the control of the Nestin promoter and conditional knockout genes in neural stem cells and as well as their derivatives including neurons, astrocytes, and oligodendrocytes. *Tia1* ^GFAP^‐CKO mice were generated by crossing the *Tia1* ^f/f^ mice with GFAP‐Cre transgenic mice (Jackson lab). These mouse strains were developed on the C57BL/6 genetic background, and their genotypes were confirmed via genotyping. The mice were allocated to various experimental groups in a random fashion. Furthermore, both genotyping and the establishment of experimental conditions were carried out in a blinded fashion. The animals were maintained in a specific pathogen‐free (SPF) environment at Hangzhou Normal University, with the temperature controlled between 18–22°C and a 12‐h light/dark cycle. They had free access to water and food. All animal experiments complied strictly with the guidelines set forth by the Laboratory Animals Ethics Committee of Hangzhou Normal University (HSD20230102).

### 2.2 EAE Model

In the present study, the experimental autoimmune encephalomyelitis (EAE) model was established following a previously reported method [22, 23]. Specifically, female mice (15–20 g), which are commonly used in published EAE studies [22], were initially anesthetized via intraperitoneal injection of 1.25% tribromoethanol at a dose of 20 mL kg^−1^. Subsequently, they received subcutaneous injections of either 200 µg of emulsified MOG35‐55 peptide (prepared in double‐distilled water, product code HY‐P1240A, MCE) or PBS, mixed in equal proportions with Complete Freund's Adjuvant (CFA) that contained 8 mg mL^−1^ of mycobacterium tuberculosis (H37RA strain, Difco, USA). Additionally, these mice were administered intraperitoneal injections of 300 ng of pertussis toxin (PTX, Sigma, dissolved in PBS) on days 0 and 2 post‐immunization. Both the injection and scoring processes were executed in a double‐blind fashion. The body weight and neurological status of the mice were assessed daily from 0 to 21 days post‐injection (dpi). Neurological assessments were based on a five‐point scoring system outlined in a previously published protocol [24]. The clinical grading of EAE symptoms was as follows: 0, no observable signs; 1, loss of tail tonicity; 2, loss of tail tonicity coupled with mild hindlimb paralysis; 3, complete hindlimb paralysis; 4, hindlimb paralysis along with mild forelimb paralysis; 5, total paralysis or death. At 21 dpi, all mice were humanely euthanized.

### 2.3 Injection of Drugs and Reagents

Diarylpropionitrile (DPN, HY‐12452, Med Chem Express) was initially dissolved in 10% DMSO and further diluted in 90% corn oil. Administration of DPN commenced one week before the induction of EAE, with mice receiving subcutaneous injections every other day at a dosage of 8 mg kg^−1^ per day [25]. Mice were randomly allocated to different experimental groups. Assessments of genotype and experimental conditions were conducted in a blinded manner.

### 2.4 Cell Culture

Primary astrocyte cultures were derived from the cerebral cortex of *Tia1* ^f/f^ and *Tia1* ^GFAP^‐CKO pups (P0–P3) following a previously established protocol [26]. In brief, the cerebral neocortex was carefully dissected and minced, then incubated in 0.125% trypsin (C0208, Beyotime) at 37°C for 15 min. Afterward, mechanical dissociation was performed to generate a single‐cell suspension by gently and repeatedly aspirating and expelling the tissue sample with a pipette. These cells were subsequently cultured in Dulbecco's Modified Eagle's Medium (DMEM, Gibco, Carlsbad, CA, USA) supplemented with 10% fetal bovine serum (FBS, Gibco) and 1% penicillin/streptomycin (Gibco, 15140‐122) under a controlled atmosphere of 95% air and 5% CO_2_ at 37°C. The isolated astrocytes were cultured on poly‐D‐lysine‐coated Petri dishes or cover glasses.

### 2.5 Hematoxylin‐Eosin (HE) Staining

Following perfusion with 0.1 M PBS and 4% paraformaldehyde (PFA), the optic nerve and retina tissues were fixed in 4% PFA for 24 h, then transferred to a 30% sucrose solution until they sank. Subsequently, the tissues were embedded in optimal cutting temperature (OCT) compound and stored at −20°C. Tissue slices of the optic nerve (5 µm thick, longitudinal) and retina (10 µm thick, cross‐section) were prepared using a Thermo CryoStar NX50 freezing microtome (Thermo Fisher Scientific, Waltham, MA, USA) and placed onto adhesive glass slides. For histological analysis, the tissue slices were colored with hematoxylin for 5 min, rinsed thoroughly in double‐distilled water, and then colored with eosin for 5 s. Drying was performed sequentially using 75%, 95%, and 100% ethanol for 30 s each. Finally, the tissue slices were made transparent in xylene for 10 min and mounted with neutral resin. Microscopic images of the colored tissue slices were recorded at room temperature using a SLIDEVIEW VS200 microscope (Olympus, Japan). Numerical evaluation of the images was carried out using ImageJ software (National Institutes of Health, Bethesda, MD, USA).

### 2.6 Nissl's Staining

The 10 µm‐thick retinal sections were subjected to incubation in a 0.1% cresyl violet solution (Solarbio) for 5 min at ambient temperature. Following this, the sections were washed with double‐distilled water and subsequently submerged in 95% ethanol for 30 s. The specimens underwent dehydration in 100% ethanol for 30 s, followed by clarification in xylene for 10 min, and were ultimately mounted using neutral resins. Microscopic imaging was conducted at room temperature using the SLIDEVIEW VS200 microscope (Olympus). The captured images were subjected to quantitative analysis using ImageJ software.

### 2.7 Luxol Fast Blue (LFB) Staining

The 5 µm‐thick optic nerve sections were subjected to an overnight staining procedure in Luxol Fast Blue (LFB) staining solution (Solarbio, G3242) at ambient temperature. After staining, the sections were washed in 95% ethanol and subsequently rinsed with distilled water. To enhance contrast, the sections were differentiated in Luxol differentiation solution for 15 s, followed by a 30‐s differentiation in 70% ethanol.

The sections were then sequentially dehydrated by immersing them in 95% and 100% ethanol, and subsequently clarified in xylene until all background color was removed. Finally, the sections were mounted using neutral resin. The extent of demyelination in the LFB‐stained sections was quantitatively evaluated using a grading system: 0 = no demyelination, 1 = moderate demyelination, and 2 = severe demyelination [27]. Microscopic observations were performed using the SLIDEVIEW VS200 microscope (Olympus), and the captured images were subjected to analysis using ImageJ software.

### 2.8 Immunostaining

Immunostaining of cultured astrocytes, 5 µm‐thick optic nerve sections, and 10 µm‐thick retinal sections was performed as previously detailed [28]. Tissues were fixed in 4% PFA for 1 day after cardiac perfusion, then dehydrated in 30% sucrose in PBS until they sank. Sections were prepared using a cryostat microtome. After three washes, optic nerve sections (5 µm, longitudinal) and retinal sections (10 µm, cross‐section) were blocked for 1 h at room temperature in 5% bovine serum albumin (BSA) and 0.3% Triton X‐100. Sections were incubated with primary antibodies overnight at 4°C, washed three times in PBS, and then incubated with secondary antibodies and DAPI in 5% BSA for 1 h at room temperature. For cultured cells, cells were washed three times with PBS, fixed in 4% PFA for 15 min, and blocked with 5% BSA and 0.1% Triton X‐100 in PBS for 60 min at room temperature. Cells were incubated with primary antibodies overnight at 4°C, washed three times in PBS, and incubated with secondary antibodies for 1 h at room temperature. Cells were then mounted after a final three PBS washes.

The primary antibodies included mouse anti‐GFAP (1:500, MAB360, Millipore), mouse anti‐Aldh1L1 (1:100, UM570039, UltraMAB), mouse anti‐MBP (1:500, ab62631, abcam), mouse anti‐NeuN (1:500, ab104224, abcam), mouse anti‐PH3(1:500, ab14955, abcam), rabbit anti‐CD45 (1:500, ab10558, abcam), rabbit anti‐NF (1:500, ab8135, abcam), rabbit anti‐TIA1 (1:500, AB140595, abcam), rabbit anti‐Iba1(1:200, ab153696, abcam), rabbit anti‐G3BP1(1:500, 13057, Proteintech), rabbit anti‐RBPMS (1:500, GTX118619, GeneTex), rabbit anti‐HMGCS1 (1:500, ab155787, Abcam). Secondary antibodies included goat anti‐mouse Alexa Fluor488 (1:1, 000, A‐11001, Thermo Fisher Scientific), goat anti‐mouse Alexa Fluor546 (1:1, 000, A‐11003, Thermo Fisher Scientific), goat anti‐rabbit Alexa Fluor546 (1:1, 000, A‐11010, Thermo Fisher Scientific), goat anti‐rabbit Alexa Fluor488 (1:1, 000, A‐11008, Thermo Fisher Scientific), goat anti‐rabbit Alexa Fluor647 (1:1, 000, A‐21244, Thermo Fisher Scientific). Microscopic imaging was performed at room temperature using a SLIDEVIEW VS200 microscope (Olympus), a confocal laser scanning microscope (FV3000RS, Olympus), or structured illumination microscopy. Image analysis was conducted using Adobe Photoshop and ImageJ software.

### 2.9 Western Blotting

Primary cultured astrocytes were collected and lysed in lysis buffer containing ice‐cold RIPA buffer (P0013b, Beyotime). Subsequently, the lysates were sonicated using an ultrasonic cell disruptor (JY‐IIN, Scientz), followed by incubation at 4°C for 20 min and then centrifugation at 14 000 × *g* at 4°C for 20 min. Mice tissues were lysed using a high‐throughput tissue grinder (Scientz) in lysis buffer. The lysate was then incubated at 4°C for 30 min, followed by centrifugation at 14 000 × *g* for 30 min at 4°C. Protein concentrations were determined using the BCA method (23227, Thermo Fisher Scientific). Subsequently, the proteins were diluted in 5× loading buffer and denatured by boiling at 100°C for 15 min. The samples were subjected to sodium dodecyl sulfate‐polyacrylamide gel electrophoresis and subsequently transferred onto polyvinylidene fluoride membranes (Pierce Chemical Company, Illinois, USA) that had been pre‐activated with methanol (HUSHI, Shanghai, China). The membranes were blocked using 1x protein‐free rapid blocking buffer for 20 min at room temperature, followed by incubation with various primary antibodies at 4°C overnight. The primary antibodies included rabbit anti‐G3BP1 (1:1, 000, 13057, Proteintech), rabbit anti‐TIA1 (1:1, 000, 12133, Proteintech), rabbit anti‐HMGCS1 (1:1, 000, ab155787, Abcam), mouse anti‐GAPDH (1:5, 000, #T0004, Affinity). Subsequently, the membranes were washed three times with TBST and then incubated with the secondary antibodies (goat anti‐mouse IgG‐HRP (1:5, 000, #31460, Pierce) and goat anti‐rabbit IgG‐HRP (1:5, 000, #31420, Pierce) for 1 h at room temperature. After incubation, the membranes were subjected to three successive washes with TBST. The protein bands were subsequently visualized utilizing an ECL detection kit (1705061, Bio‐Rad, USA). The intensity of the bands was quantified by employing ImageJ software.

### 2.10 Electron Microscopy

We followed a previously described protocol for electron microscopy [22]. The tissues were fixed overnight in 2.5% glutaraldehyde (sourced from Guoyao Chemical Reagent Co., Ltd.). Subsequent to fixation, the tissues were subjected to PBS washes and a secondary fixation in 1% osmium tetroxide (SPI‐CHEM). Following additional PBS washes, the tissues were dehydrated using a graded series of alcohol solutions. Subsequently, the tissues were immersed in absolute acetone (Sinopharm Chemical Reagent Co., Ltd.) and embedded in Spurr resin (SPI‐CHEM) overnight. After heating the samples at 70°C for over 9 h, they were sectioned using a LEICA EM UC7 ultra‐thin microtome. The sections were subsequently stained with uranyl acetate and alkaline lead citrate (Sinopharm Chemical Reagent Co., Ltd.). Finally, images were acquired using a Hitachi H‐7650 Transmission Electron Microscope (TEM).

### 2.11 Quantitative Reverse Transcription Polymerase Chain Reaction (qRT‐PCR)

RNA extraction was performed with the RNA‐Quick Purification Kit (YISHAN Biotechnology, ES‐RN001) in accordance with the manufacturer's instructions. The quantification of RNA was performed using the Thermo Scientific NanoDrop One. Subsequently, cDNA was synthesized using the cDNA DyNAmo Kit (Vazyme, R211‐01/02) was utilized. The qRT‐PCR analysis was conducted using the SYBR Green PCR master mix (Vazyme Biotech, Q511‐02/03). The real‐time PCR cycling parameters were set as follows: an initial step at 95°C for 15 min, followed by 40 cycles of denaturation at 94°C for 15 s, annealing at 56°C or 60°C for 30 s, and a final extension at 72°C for 30 s. Following amplification, a melting curve analysis was conducted to validate primer specificity. GAPDH was used as the endogenous control for data normalization. Primers used were *HMGCS1*‐F: 5′ TGT TCT CTT ACG GTT CTG GC 3′, *HMGCS1*‐R: 5′ AAG TTC TCG AGT CAA GCC TTG 3′; *FDPS*‐F: 5′ AGG AGG TCC TAG AGT ACA ATG CC 3′, *FDPS*‐R: 5′ TGA GGG AAG AGT CCA TGA TGT C 3′; *SQLE*‐F: 5′ ACC CGG AAG TGA TCA TCG TG 3′, *SQLE*‐R: 5′ ATG GGC ATT GAG ACC TTC TAC TGT 3′; *Ldlr*‐F: 5′ ACC CGC CAA GAT CAA GAA AG 3′, *Ldlr*‐R: 5′ GCT GGA GAT AGA GTG GAG TTT G 3′; *HMGCR*‐F: 5′ AGT CAG TGG GAA CTA TTG CAC 3′, *HMGCR*‐R: 5′ TTA CGT CAA CCA TAG CTT CCG 3′; *GAPDH*‐F: 5′ AGG TCG GTG TGA ACG GAT TTG 3′, *GAPDH*‐R: 5′ TGT AGA CCA TGT AGT TGA GGT CA‐3′.

### 2.12 Fluorescence In Situ Hybridization (FISH)

RNA‐FISH was conducted using a Ribo fluorescence in situ hybridization kit (C10910, RiboBio) in accordance with the manufacturer's directions. Briefly, cells were seeded and fixed with 4% PFA, then permeabilized with 0.5% Triton X‐100 in PBS, and subsequently subjected to pre‐hybridization. The fixed cells were hybridized overnight at a probe concentration of 0.5µM. The HMGCS1 FISH probes were designed and synthesized by RiboBio. All images were visualized and obtained by a confocal laser scanning microscope (FV3000RS, Olympus).

### 2.13 RNA Immunoprecipitation

RNA immunoprecipitation (RIP) experiments were conducted using an RNA Immunoprecipitation Kit (BersinBio, Bes5101) to further confirm the binding relationship between *HMGCS1* and proteins. In this study, a TIA1 antibody (AB140595, Abcam) was used to target and capture RNA molecules binding to the TIA1 proteins. Briefly, according to the manufacturer's instructions, the optic nerves of EAE mice were collected and fully lysed in polysome lysis buffer. Then, the tissue lysate was divided into two groups (labeled as IgG and TIA1), and 6 µg of the IgG protein (negative control) and TIA1 antibody were added. The antibody‐containing cell lysate was mixed vertically at 4°C for 16 h to allow the antibody to capture the corresponding protein. Then, Protein A/G beads were used to collect the target protein and the bound RNA molecules. Real‐time qPCR was performed to evaluate the mRNA of the target gene. Primers used were *HMGCS1*‐F: 5′ TGT TCT CTT ACG GTT CTG GC 3′, *HMGCS1*‐R: 5′ AAG TTC TCG AGT CAA GCC TTG 3′.

### 2.14 RNA Sequencing and Functional Enrichment Analysis

The RNA sequencing was performed as previous report [22]. The Novogene Bioinformatics Institute (Beijing, China) was responsible for RNA sequencing. Total RNA extraction from the optic nerve of *Tia1* ^Nestin^‐CKO and *Tia1* ^f/f^ EAE mice was performed using Trizol (Invitrogen) in accordance with the manufacturer's instructions. RNA purity was assessed using Nanodrop, with an A260:A280 ratio exceeding 1.8 and an A260:A230 ratio exceeding 2.0 for each sample. RNA integrity and quantity were further assessed using the RNA Nano 6000 Assay Kit on the Bioanalyzer 2100 system (Agilent Technologies, CA, USA). Library fragments were purified using the AMPure XP system (Beckman Coulter, Beverly, USA), with cDNA fragments of 370–420 bp selected. Subsequent PCR amplification and purification using AMPure XP beads produced the final library. Initial library quantification was performed using the Qubit 2.0 Fluorometer, with the library diluted to 1.5 ng µL^−1^, and insert size detection carried out using the Agilent 2100 bioanalyzer. qRT‐PCR was employed to ensure accurate quantification of library concentration. Sequencing employed the Illumina NovaSeq 6000, generating 150 bp paired‐end reads.

Clean data (clean reads) were generated by eliminating reads containing adapters, reads with N bases, and low‐quality reads from the raw data. The reference genome index was constructed using Hisat2 (v2.0.5), and paired‐end clean reads were aligned to the reference genome using Hisat2. FeatureCounts (v1.5.0‐p3) was subsequently used to count the number of reads mapped to each gene, and the Fragments Per Kilobase of transcript sequence per Millions base pairs sequenced (FPKM) for each gene was calculated. Differential expression analysis between the two groups was conducted using the DESeq2 R package (1.20.0), with a *p*‐value threshold of less than 0.05 for statistical significance. Gene Ontology (GO) enrichment analysis was based on differentially expressed genes (DEGs) with a *p*‐value less than 0.05.

### 2.15 Filipin Staining

Filipin staining analysis was conducted to examine free cholesterol in optic nerves. The sections were fixed in 4% paraformaldehyde for 30 min, after which the samples were washed with PBS. Filipin dye solution (GLPBIO, GC12048, California, USA) was added, and the samples were stained in the dark for 2 h at room temperature. Microscopic imaging was performed at room temperature using a confocal laser scanning microscope. Image analysis was conducted using ImageJ software.

### 2.16 Total Cholesterol (TC) Assay

Mouse optic nerves were harvested and processed on ice. The tissues were directly homogenized in physiological saline (0.9% NaCl). According to the manufacturer's protocol, total cholesterol was quantified using a total cholesterol assay kit (SC31N0200, Wuhan Szy Biotech Co., Ltd., China). The samples were analyzed using a fully automated biochemical analyzer (BS‐370E, Shenzhen Mindray Bio‐Medical Electronics Co., Ltd., China).

### 2.17 Data Analysis and Statistics

All data values were expressed as mean ± SEM derived from at least three independent experiments. Statistical analysis was done with GraphPad Prism and ImageJ software. Student's t‐test was used for comparison between two groups, and two‐way ANOVA with Tukey's multiple comparisons test was performed for multiple groups. A *p*‐value of <0.05 was statistically significant.

## 3 Results

### 3.1 TIA1 was Upregulated in Retinal Neurons and Optic Nerve Astrocytes of EAE Mice

To explore potential roles of TIA1‐mediated SGs in MS‐ON, EAE, a common typical model of MS, was established. C57BL/6 female mice (8–10 weeks) were immunized with MOG35‐55 and a pertussis toxin boost. As expected, these mice developed an acute monophasic EAE, and at 21 dpi, the optic nerve and retinal tissues of EAE mice were collected. Then myelin basic protein (MBP) (a marker of myelin) immunostaining showed the obvious demyelination in the optic nerves of EAE mice (Figure S1A,B). Furthermore, immunostaining of Iba1 (a marker of microglia) and GFAP (a marker of astrocytes) also showed the obvious activation of microglia and astrocytes, and inflammatory infiltration in optic nerves and retina of EAE mice (Figure S1C–G). These results indeed indicated that optic neuritis of the EAE model was established successfully.

**FIGURE 1:**
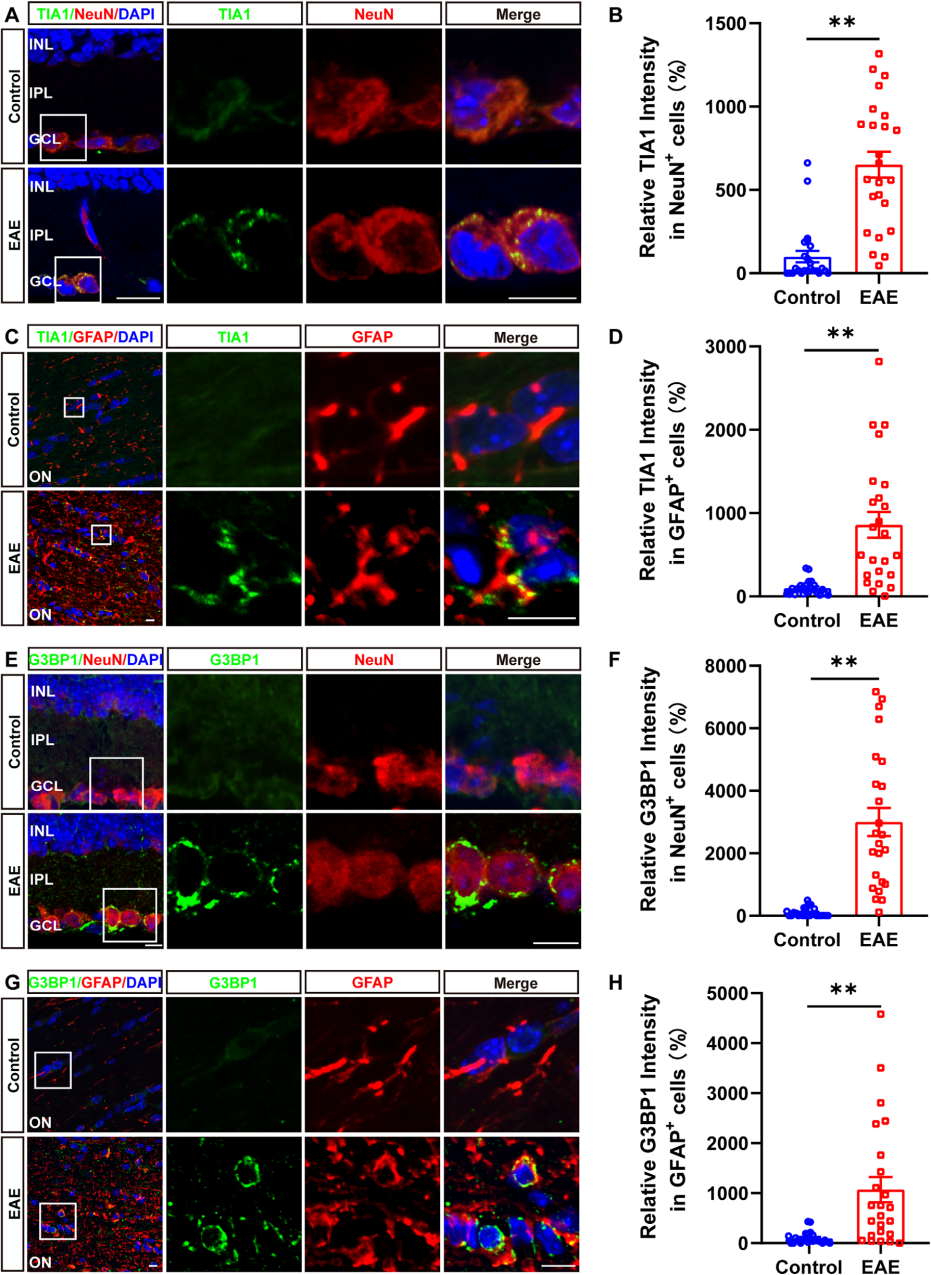
TIA1 was upregulated in retinal neurons and optic nerve astrocytes of EAE mice. (A) Double immunostaining of TIA1 (green) and NeuN (red) in the retina of control and EAE mice. (B) Quantitative analysis of the relative TIA1 intensity as shown in (A) (n = 6, normalized to control). (C) Double immunostaining of TIA1 (green) and GFAP (red) in the optic nerves of control and EAE mice. (D) Quantitative analysis of the relative TIA1 intensity as shown in (C) (n = 6, normalized to control). (E) Double immunostaining of G3BP1 (green) and NeuN (red) in the retina of control and EAE mice. (F) Quantitative analysis of the relative G3BP1 intensity as shown in (E) (n = 6, normalized to control). (G) Double immunostaining of G3BP1 (green) and GFAP (red) in the optic nerves of control and EAE mice. (H) Quantitative analysis of the relative G3BP1 intensity as shown in (G) (n = 6, normalized to control). Scale bars, 10 µm. ^*^p < 0.05, ^**^p < 0.01.

Next, we examined the expression level and the cellular expression pattern of TIA1 and SGs in optic nerves and retina of EAE mice. As shown in Figure 1A–H, the expression level of TIA1 and G3BP1 (a cell marker of SGs) was significantly upregulated in retinal NeuN^+^ neurons and optic nerve GFAP^+^ astrocytes of EAE mice. Taken together, these results suggested that TIA1 and G3BP1^+^ SGs were upregulated in retinal neurons and optic nerve astrocytes of EAE mice.

### 3.2 The Demyelination was Alleviated in Optic Nerves of Tia1^Nestin^‐CKO EAE Mice

To further investigate the roles of neuronal and astrocytic TIA1 in MS‐ON, CNS‐specific conditional *Tia1* knockout mice (*Tia1* ^Nestin^‐CKO) were generated by crossing *Tia1* ^f/f^ mice with Nestin‐Cre transgenic mice (Figure S2A,B), resulting in conditional ablation of *Tia1* in neural stem cells and their derivatives, including neurons, astrocytes, and oligodendrocytes. Indeed, the expression of TIA1 was significantly reduced in the brain regions of *Tia1* ^Nestin^‐CKO mice, such as the cortex, olfactory bulb, cerebellum, and spinal cord (Figure S2C,D). Subsequently, HE staining showed that *Tia1* deletion in the CNS did not significantly affect the development of optic nerves (Figure S3A,B). Moreover, immunostaining of GFAP and Iba1 showed that the distribution and number of astrocytes and microglia were not affected in the optic nerves of *Tia1* ^Nestin^‐CKO mice (Figure S3C–F). Consistent with these results shown in optic nerves, immunostaining of RBPMS (a marker of RGCS), NeuN, GFAP, and Iba1 in retina also showed that there were no significant differences in the distribution and number of RGCS, astrocytes, and microglia between *Tia1* ^f/f^ and *Tia1* ^Nestin^‐CKO mice (Figure S3G–N). Taken together, these results suggested that *Tia1* deletion in the CNS did not affect the normal development of optic nerves and retina in mice.

**FIGURE 2:**
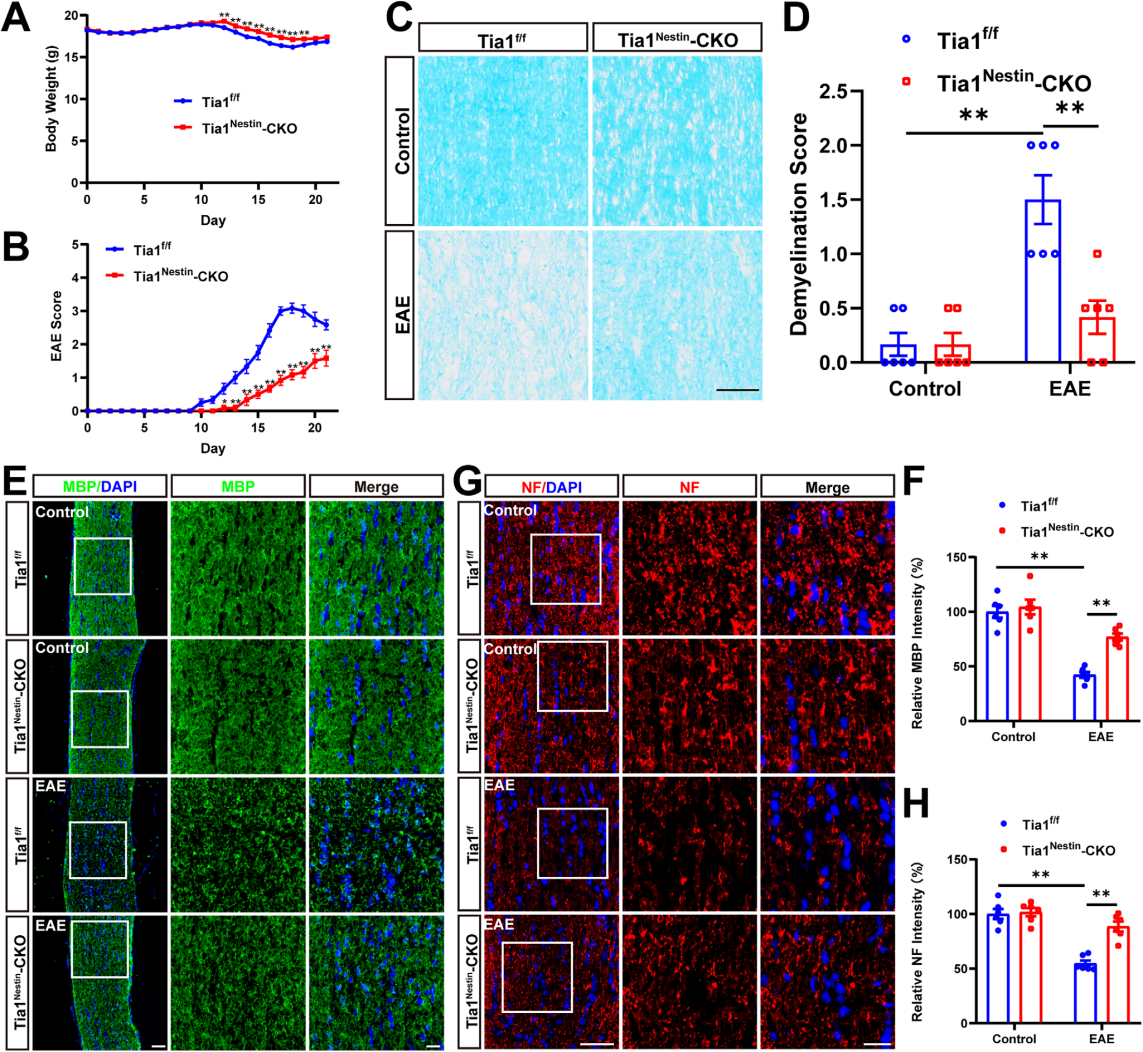
The demyelination was alleviated in optic nerves of *Tia1* ^Nestin^‐CKO EAE mice. (A) The body weight of *Tia1* ^f/f^ and *Tia1* ^Nestin^‐CKO mice from 0 to 21 dpi during the process of EAE modeling (n = 6). (B) The EAE score of *Tia1* ^f/f^ and *Tia1* ^Nestin^‐CKO mice 0 to 21 dpi during the process of EAE modeling (n = 6). (C) LFB staining of optic nerves from *Tia1* ^f/f^ and *Tia1* ^Nestin^‐CKO mice, *Tia1* ^f/f^ EAE and *Tia1* ^Nestin^‐CKO EAE mice. (D) Quantitative analysis of the demyelination score as shown in (C) (n = 6). (E) Immunostaining of MBP (green) in the optic nerves from *Tia1* ^f/f^ and *Tia1* ^Nestin^‐CKO mice, *Tia1* ^f/f^ EAE and *Tia1* ^Nestin^‐CKO EAE mice. (F) Quantitative analysis of the relative MBP intensity as shown in (E) (n = 6, normalized to *Tia1* ^f/f^ mice). (G) Immunostaining of NF (red) in the optic nerves from *Tia1* ^f/f^ and *Tia1* ^Nestin^‐CKO mice, *Tia1* ^f/f^ EAE and *Tia1* ^Nestin^‐CKO EAE mice. (H) Quantitative analysis of the relative NF intensity as shown in (G) (n = 6, normalized to *Tia1* ^f/f^ mice). Images of selected regions (white squares) were shown at higher magnification. Scale bars, 50  and 20 µm (enlarge). ^*^p < 0.05, ^**^p < 0.01.

*Tia1* ^f/f^ and *Tia1* ^Nestin^‐CKO mice were next injected with MOG_35‐55_ and pertussis toxin to induce EAE. As shown in Figure 2A, there was a significant difference in body weight between *Tia1* ^f/f^ EAE and *Tia1* ^Nestin^‐CKO EAE mice. Furthermore, *Tia1* ^Nestin^‐CKO EAE mice showed a delayed onset of initial symptoms (characterized by tail paralysis) and a substantially reduced EAE score (Figure 2B). To exclude the possibility that this difference was due to intrinsic developmental defects, we compared the body weights of *Tia1* ^f/f^ and *Tia1* ^Nestin^‐CKO mice and found no significant differences in their growth curves [22]. Extended evaluation of EAE score through day 23 revealed that *Tia1* ^Nestin^‐CKO EAE mice exhibited a lower peak EAE score compared with *Tia1* ^f/f^ EAE mice (Figure S4A). These findings suggest that dyskinesia of EAE was alleviated in *Tia1* ^Nestin^‐CKO EAE mice, rather than merely reflecting a delay in disease onset. Consistent with the EAE score, the body weights of *Tia1* ^Nestin^‐CKO EAE mice also showed signs of recovery and stabilization in the later phase (Figure S4B). It has been shown that the severity of optic nerve histopathology is positively correlated with clinical EAE scores by assessing the severity of infiltration and demyelination of inflammatory cells in the optic nerves [27]. As expected, LFB staining and MBP immunostaining in optic nerves indeed showed that there was a significant decrease in demyelination score and a significant increase in the intensity of MBP in *Tia1* ^Nestin^‐CKO EAE mice (Figure 2C–F). The similar results of the neurofilament heavy polypeptide (NF) intensity were observed in *Tia1* ^Nestin^‐CKO EAE mice (Figure 2G,H). Taken together, these results indicated that *Tia1* deletion in the CNS alleviated the demyelination in the optic nerves of EAE mice.

### 3.3 The Inflammatory Infiltration Was Relieved in Optic Nerves of Tia1^Nestin^‐CKO EAE Mice

**FIGURE 3:**
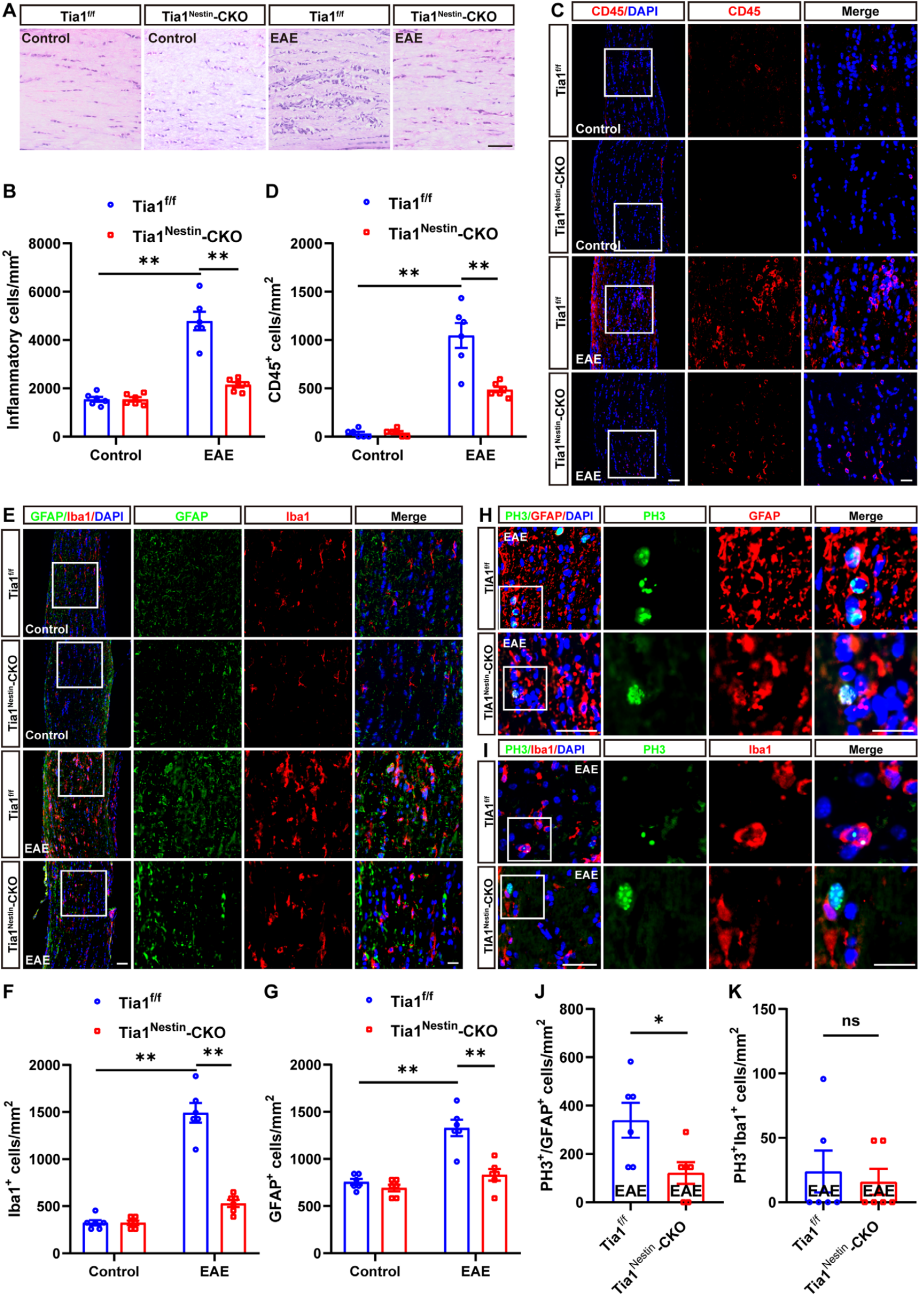
The inflammatory infiltration was relieved in optic nerves of *Tia1* ^Nestin^‐CKO EAE mice. (A) HE staining of optic nerves from *Tia1* ^f/f^ and *Tia1* ^Nestin^‐CKO mice, *Tia1* ^f/f^ EAE and *Tia1* ^Nestin^‐CKO EAE mice. (B) Quantitative analysis of the density of inflammatory cells as shown in (A) (n = 6). (C) Immunostaining of CD45 (red) in the optic nerves from *Tia1* ^f/f^ and *Tia1* ^Nestin^‐CKO mice, *Tia1* ^f/f^ EAE and *Tia1* ^Nestin^ ‐CKO EAE mice. (D) Quantitative analysis of the density of CD45^+^ cells as shown in (C) (n = 6). (E) Double immunostaining of Iba1 (red) and GFAP (green) in the optic nerves from *Tia1* ^f/f^ and *Tia1* ^Nestin^‐CKO mice, *Tia1* ^f/f^ EAE and *Tia1* ^Nestin^‐CKO EAE mice. (F‐G) Quantitative analysis of the density of Iba1^+^ cells (F) or GFAP^+^ cells (G) as shown in (E) (n = 6). (H‐I) Double immunostaining of PH3 (green) and GFAP (red) (H), or PH3 (green) and Iba1 (red) (I) in the optic nerves from *Tia1* ^f/f^ EAE and *Tia1* ^Nestin^‐CKO EAE mice. (J‐K) Quantitative analysis of density of PH3^+^/GFAP^+^ cells (J) or PH3^+^/Iba1^+^ cells (K) as shown in (H) or (I) (n = 6), respectively. Images of selected regions (white squares) were shown at higher magnification. Scale bars, 50  and 20 µm (enlarge). ^*^p < 0.05, ^*^p < 0.01; n.s., not significant.

Recent studies have suggested that inflammatory infiltration has a tight connection with demyelination in EAE mice [29]. Indeed, as shown in Figure 3A,B, HE staining showed that the inflammatory infiltration was obviously relieved in optic nerves of *Tia1* ^Nestin^‐CKO EAE mice, compared with that in *Tia1* ^f/f^ EAE mice. As inflammatory cells under ON conditions include astrocytes, microglia cells, and T cells, interestingly, the density of CD45^+^ (a marker of T cells) cells was also decreased in optic nerves of *Tia1* ^Nestin^‐CKO EAE mice (Figure 3C,D). Meanwhile, we also found that GFAP^+^ astrocytes and Iba1^+^ microglia displayed a hypertrophic morphology (activated phenotype) in *Tia1* ^f/f^ EAE mice; however, the activation of microglia and astrocytes was inhibited, and the density of these cells was also reduced in optic nerves of *Tia1* ^Nestin^‐CKO EAE mice (Figure 3E–G), suggesting that *Tia1* deletion in the CNS not only decreased the number of T cells, but also suppressed the activation of astrocytes and microglia in optic nerves of EAE mice. Furthermore, as shown in Figure 3H–K, PH3 (phospho‐histone H3) immunostaining, a marker of proliferating cells, indicated that astrocyte proliferation—but not microglial proliferation—was decreased in the optic nerves of *Tia1* ^Nestin^‐CKO EAE mice, compared with that in *Tia1* ^f/f^ EAE mice. Taken together, these results indicated that the inflammatory infiltration was relieved and the proliferation of astrocytes was inhibited in the optic nerves of *Tia1* ^Nestin^‐CKO EAE mice.

### 3.4 The Loss of RGCs and the Inflammatory Infiltration Were Relieved in the Retina of Tia1^Nestin^‐CKO EAE Mice

**FIGURE 4:**
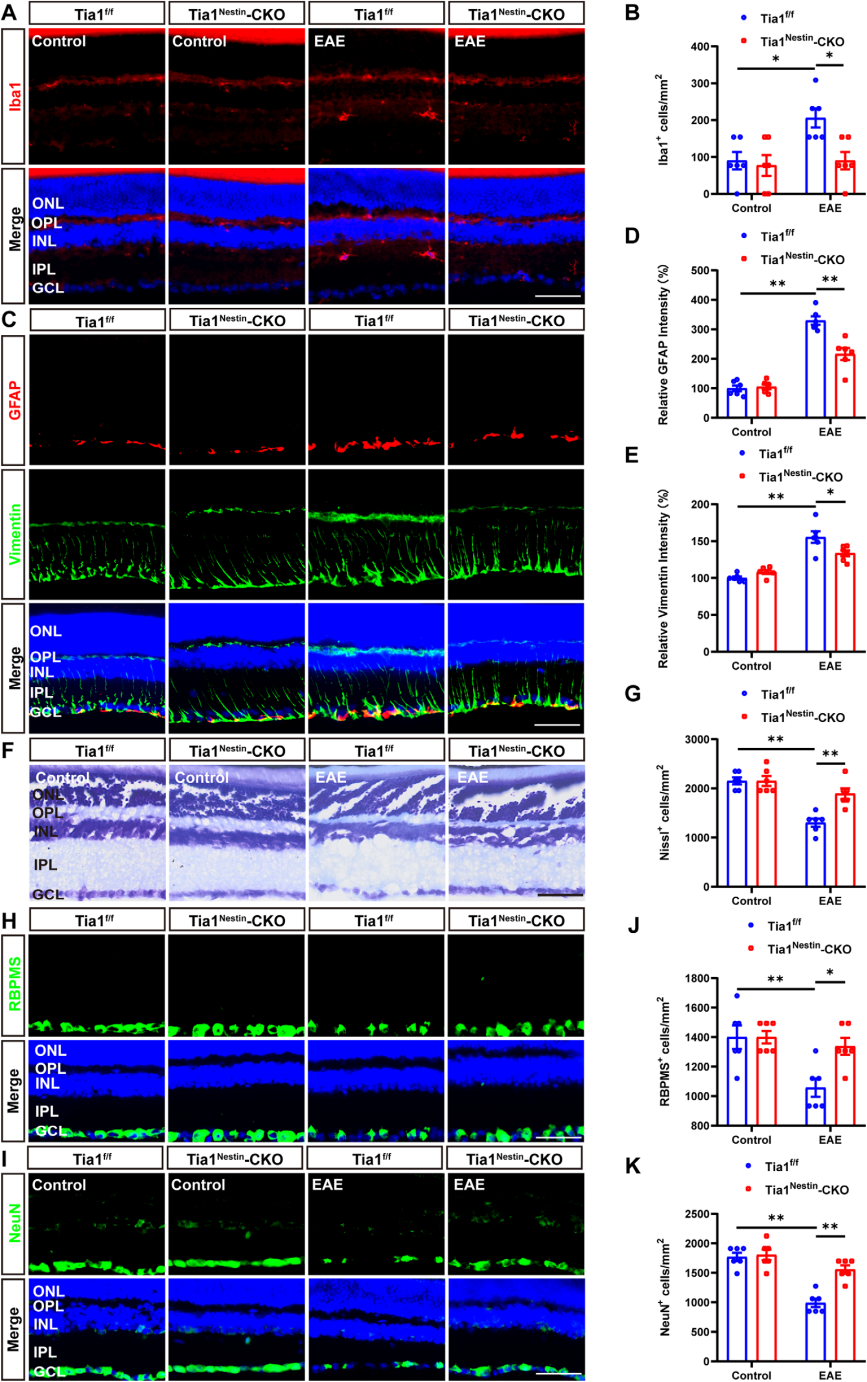
The loss of RGCs and the inflammatory infiltration were reduced in retina of *Tia1* ^Nestin^‐CKO EAE mice. (A) Immunostaining of Iba1 (red) in the retina from *Tia1* ^f/f^ and *Tia1* ^Nestin^‐CKO mice, *Tia1* ^f/f^ EAE and *Tia1* ^Nestin^‐CKO EAE mice. (B) Quantitative analysis of the density of Iba1^+^ cells as shown in (A) (n = 6). (C) Double immunostaining of GFAP (red) and vimentin (green) in the optic nerves from *Tia1* ^f/f^ and *Tia1* ^Nestin^‐CKO mice, *Tia1* ^f/f^ EAE and *Tia1* ^Nestin^‐CKO EAE mice. (D‐E) Quantitative analysis of the relative GFAP intensity (D) or vimentin intensity (E) as shown in (C) (n = 6, normalized to *Tia1* ^f/f^ mice). (F) Representative images of Nissl staining in the retina of *Tia1* ^f/f^ and *Tia1* ^Nestin^‐CKO mice, *Tia1* ^f/f^ EAE and *Tia1* ^Nestin^‐CKO EAE mice. (G) Quantitative analysis of the density of Nissl^+^ cells in the ganglion cell layer as shown in (F) (n = 6). (H,I) Immunostaining of RBPMS (green) (H) or NeuN (green) (I) in the retina of *Tia1* ^f/f^ and *Tia1* ^Nesti^ *^n^*‐CKO mice, *Tia1* ^f/f^ EAE and *Tia1* ^Nestin^‐CKO EAE mice. (J,K) Quantitative analysis of the density of RBPMS^+^ cells (J) or NeuN^+^ cells (K) as shown in (H) or (I), respectively (n = 6). Scale bars, 50 µm. ^*^p < 0.05, ^**^p < 0.01.

Inflammatory infiltration in optic neuritis is found not only in the optic nerves, but also in the retina [30]. As expected, there was a significant decrease in the density of Iba1^+^ microglia and the intensity of GFAP^+^ or vimentin^+^ (a marker of reactive astrocytes) astrocytes in the retina of *Tia1* ^Nestin^‐CKO EAE mice, compared with that in *Tia1* ^f/f^ EAE mice (Figure 4A–E). Recent studies have shown that the inflammatory infiltration in ON is associated with RGCs loss to some extent [31]. Nissl staining indeed showed that the loss of RGCs was significantly inhibited in retina of *Tia1* ^Nestin^‐CKO EAE mice, compared with that in *Tia1* ^f/f^ EAE mice (Figure 4F,G). Consistently, as shown in Figure 4H–K, the loss of RBPMS^+^ or NeuN^+^ RGCs (both RBPMS and NeuN are markers of RGCs) was also significantly inhibited in the retina of *Tia1* ^Nestin^‐CKO EAE mice, compared with that in *Tia1* ^f/f^ EAE mice. Taken together, these results suggested that *Tia1* deletion in the CNS relieved the inflammatory infiltration in the retina, which resulted into less RCGs loss in the retina of EAE mice.

### 3.5 The Cholesterol‐Synthesis Pathway Genes Were Upregulated in Tia1^Nestin^‐CKO EAE Mice

**FIGURE 5:**
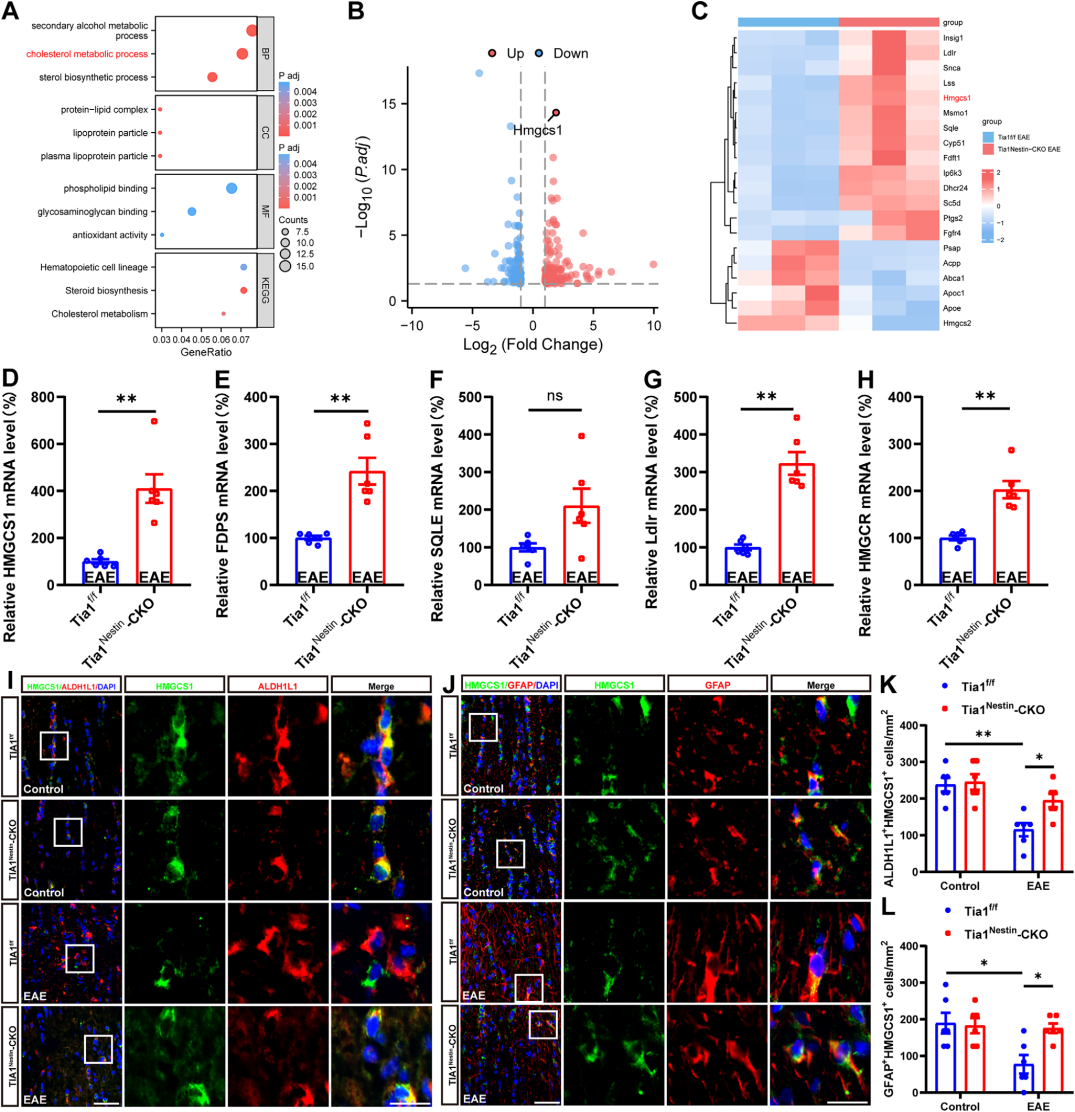
The cholesterol‐synthesis pathway genes were upregulated in optic nerves of *Tia1* ^Nestin^‐CKO EAE mice. (A) GO enrichment analysis of differential mRNAs in optic nerves of *Tia1* ^f/f^ EAE and *Tia1* ^Nestin^‐CKO EAE mice (n = 3). (B,C) The volcano plot (B) and heatmap (C) of differential mRNAs in optic nerves of *Tia1* ^f/f^ EAE and *Tia1* ^Nestin^‐CKO EAE mice (n = 3). (D–H) qRT‐PCR analysis of the relative mRNA levels of *HMGCS1* (D), *FDPS* (E), *SQLE* (F), *Ldlr* (G), and *HMGCR* (H) in the optic nerves of *Tia1* ^f/f^ EAE and *Tia1* ^Nestin^‐CKO EAE mice (n = 6, normalized to *Tia1* ^f/f^ EAE mice). (I,J) Double immunostaining of HMGCS1 (green) and ALDH1L1 (red) (I), or HMGCS1 (green) and GFAP (red) (J) in the optic nerves of *Tia1* ^f/f^ and *Tia1* ^Nestin^‐CKO mice, *Tia1* ^f/f^ EAE and *Tia1* ^Nestin^‐CKO EAE mice. (K,L) Quantitative analysis of density of ALDH1L1^+^/HMGCS1^+^ cells (K) or GFAP^+^/HMGCS1^+^ cells (L) as shown in (I) or (J), respectively (n = 6). Scale bars, 50 and 20 µm (enlarge). ^*^p < 0.05, ^**^p < 0.01.

How does *Tia1* deletion in the CNS alleviate the demyelination in the optic nerves of EAE mice? For this purpose, mRNA sequencing of optic nerves from *Tia1* ^f/f^ EAE mice and *Tia1* ^Nestin^‐CKO EAE mice was carried out (Figure 5A–C). Interestingly, genes of the cholesterol‐synthesis pathway, such as *HMGCS1, LSS, SQLE, and FDFT1* were significantly upregulated in optic nerves of *Tia1* ^Nestin^‐CKO EAE mice. These genes have been reported to be involved in reparative synaptic plasticity and myelination in EAE mice [32]. qRT‐PCR analysis further confirmed the up‐regulation of the mRNA level of *HMGCS1, HMGCR, LDLR*, and *FDPS* in *Tia1* ^Nestin^‐CKO EAE mice, while SQLE showed an upward trend that did not reach statistical significance (Figure 5D–H). It has been reported that HMGCS1 is an important factor for cholesterol biosynthesis [33]. At the protein level, double immunostaining of HMGCS1/ALDH1L1 and HMGCS1/GFAP showed the obvious up‐regulation of HMGCS1 in optic nerve astrocytes of *Tia1* ^Nestin^‐CKO EAE mice (Figure 5I–L). Taken together, these results suggested that the cholesterol‐synthesis pathway genes were upregulated in *Tia1* ^Nestin^‐CKO EAE mice, and these genes, such as *HMGCS1* mRNA, might be selectively sequestered into TIA1‐mediated SGs, which might promote the demyelination in EAE mice.

### 3.6 The Demyelination and the Inflammatory Infiltration Were Alleviated in the Optic Nerves of Tia1^GFAP^‐CKO EAE Mice

**FIGURE 6:**
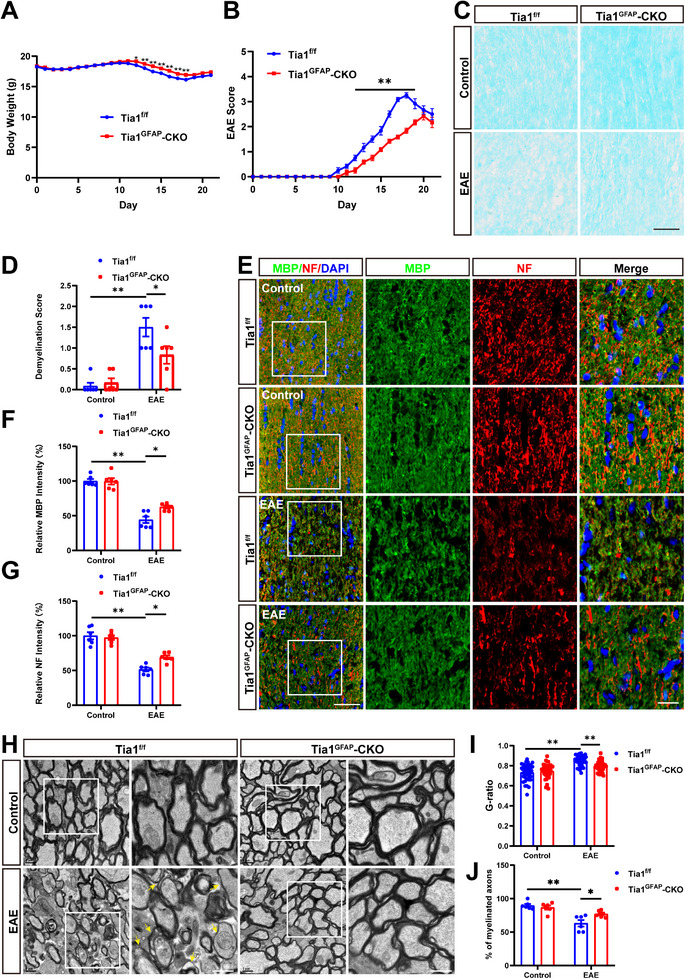
The demyelination was alleviated in optic nerves of *Tia1* ^GFAP^‐CKO EAE mice. (A) The body weight of *Tia1* ^f/f^ and *Tia1* ^GFAP^‐CKO mice from 0 to 21 dpi during the process of EAE modeling (n = 6). (B) The EAE score of *Tia1* ^f/f^ and *Tia1* ^GFAP^‐CKO mice 0 to 21 dpi during the process of EAE modeling (n = 6). (C) LFB staining of optic nerves from *Tia1* ^f/f^ and *Tia1* ^GFAP^‐CKO mice, *Tia1* ^f/f^ EAE and *Tia1* ^GFAP^‐CKO EAE mice. (D) Quantitative analysis of the demyelination score as shown in (C) (n = 3). (E) Double immunostaining of MBP (green) and NF (red) in the optic nerves of *Tia1* ^f/f^ and *Tia1* ^GFAP^‐CKO mice, *Tia1* ^f/f^ EAE and *Tia1* ^GFAP^‐CKO EAE mice. (F,G) Quantitative analysis of the relative MBP intensity (F) or NF intensity (G) as shown in (E) (n = 6, normalized to *Tia1* ^f/f^ mice). (H) The typical electron microscopic images in the optic nerves of *Tia1* ^f/f^ and *Tia1* ^GFAP^‐CKO mice, *Tia1* ^f/f^ EAE and *Tia1* ^GFAP^‐CKO EAE mice at 21 dpi. Scale bars, 1 µm. (I) Quantification of G‐ratio as shown in (H) (n = 42). (J) Quantification of the percentages of myelinated axons in one field, as shown in (H) (n = 6). Scale bars, 50 and 20 µm (enlarge). ^*^p < 0.05, ^**^p < 0.01.

It has been reported that cholesterol synthesis gene expression is decreased in optic nerve astrocytes during EAE, especially HMGCS1 [32]. To further investigate the role of astrocytic TIA1 in MS‐ON, conditional *Tia1* knockout mice in astrocytes were generated through crossing *Tia1* ^f/f^ with GFAP‐cre transgenic mice (*Tia1* ^GFAP^‐CKO mice), which conditionally ablation of *Tia1* specifically in astrocytes (Figure S5A–C). *Tia1* ^f/f^ and *Tia1* ^GFAP^‐CKO mice were next injected with MOG35‐55 and pertussis toxin to induce EAE. As shown in Figure 6A, there was a difference in body weight between *Tia1* ^f/f^ EAE and *Tia1* ^GFAP^‐CKO EAE mice. However, deletion of *Tia1* in astrocytes also showed better clinical scores (Figure 6B). As expected, LFB staining and MBP immunostaining in optic nerves indeed showed that there was a significant decrease in demyelination score and an increase in MBP intensity of *Tia1* ^GFAP^‐CKO EAE mice (Figure 6C–F). These findings were further confirmed by NF immunostaining (Figure 6E–G). Furthermore, EM experiment further showed that *Tia1* ^GFAP^‐CKO EAE mice had less demyelination phenotype in optic nerves than that in *Tia1* ^f/f^ EAE mice (Figure 6H). The G‐ratio and the percentage of myelinated axons were significantly decreased in *Tia1* ^f/f^ EAE mice; however, these decreases were significantly inhibited in *Tia1* ^GFAP^‐CKO EAE mice (Figure 6I,J). Taken together, these results suggested that *Tia1* deletion in astrocytes alleviated the demyelination in optic nerves of EAE mice.

**FIGURE 7:**
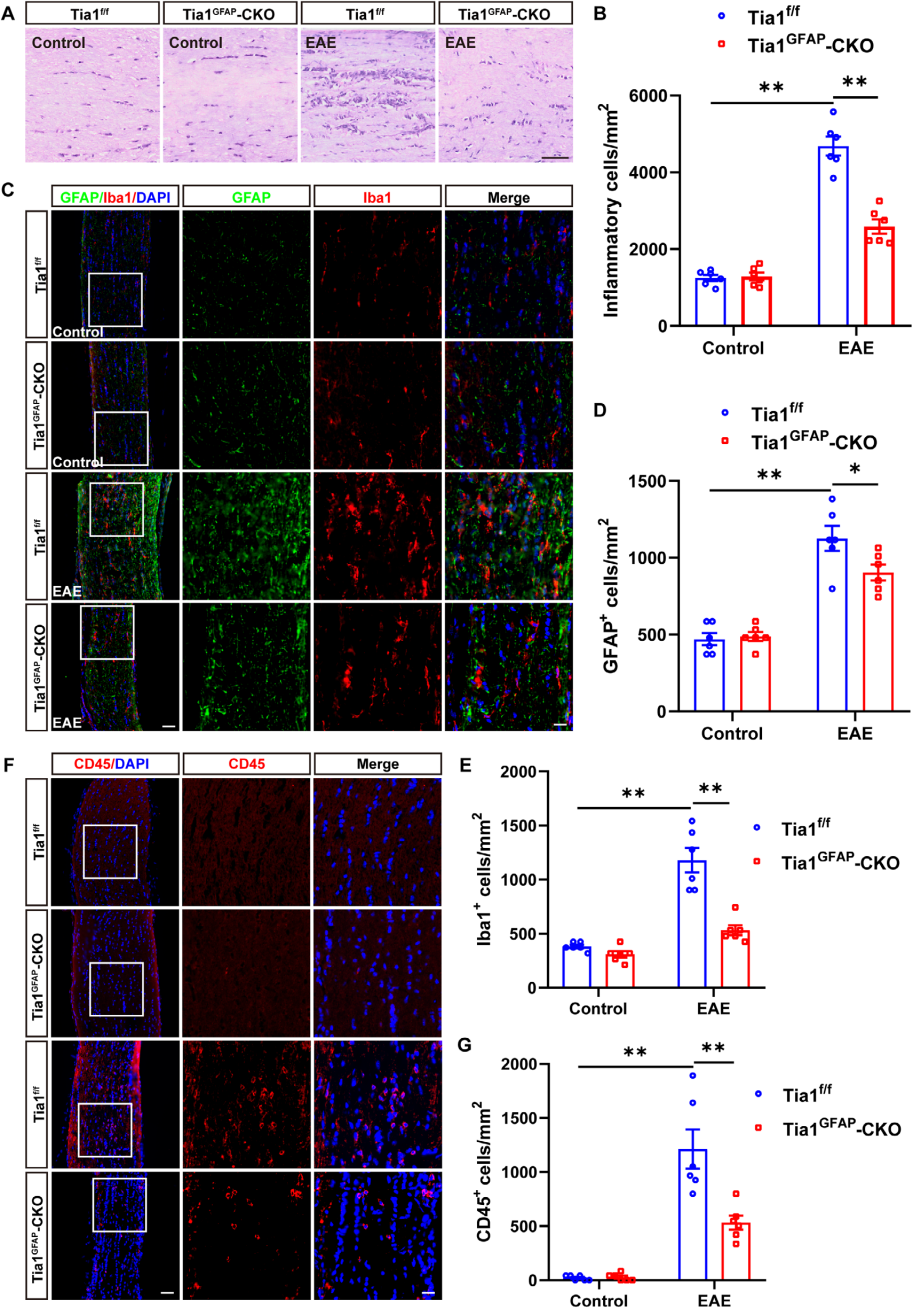
The inflammatory infiltration was relieved in optic nerves of *Tia1* ^GFAP^‐CKO EAE mice. (A) HE staining of optic nerves from *Tia1* ^f/f^ and *Tia1* ^GFAP^‐CKO mice, *Tia1* ^f/f^ EAE, and *Tia1* ^GFAP^‐CKO EAE mice. (B) Quantitative analysis of the density of inflammatory cells as shown in (A) (n = 6). (C) Double immunostaining of GFAP (green) and Iba1 (red) in the optic nerves of *Tia1* ^f/f^ and *Tia1* ^GFAP^‐CKO mice, *Tia1* ^f/f^ EAE and *Tia1* ^GFAP^ ‐CKO EAE mice. (D) Quantitative analysis of the relative GFAP^+^ cells as shown in (C) (n = 6, normalized to *Tia1* ^f/f^ mice). (E) Quantitative analysis of the density of Iba1^+^ cells as shown in (C) (n = 6). (F) Immunostaining of CD45 (red) in the optic nerves from *Tia1* ^f/f^ and *Tia1* ^GFAP^‐CKO mice, *Tia1* ^f/f^ EAE, and *Tia1* ^GFAP^‐CKO EAE mice. (G) Quantitative analysis of the density of CD45^+^ cells as shown in (F) (n = 6). Scale bars, 50 and 20 µm (enlarge). ^*^p < 0.05, ^**^p < 0.01.

To examine whether *Tia1* deletion in astrocytes would affect the inflammatory infiltration, HE staining was performed in EAE mice. As shown in Figure 7A,B, inflammatory infiltration in the optic nerves of *Tia1* ^GFAP^‐CKO EAE mice was relieved obviously. Meanwhile, the activation of astrocytes and microglia was suppressed in *Tia1* ^GFAP^‐CKO EAE mice (Figure 7C–E). The density of CD45^+^ cells was also decreased in optic nerves of *Tia1* ^GFAP^‐CKO EAE mice (Figure 7F,G), suggesting that *Tia1* deletion in astrocytes not only suppressed astrocytic and microglia activation, but also decreased the number of T cells in optic nerves of EAE mice. Taken together, these results suggested that astrocytic *Tia1* deletion relieved the inflammatory infiltration in the optic nerves of EAE mice.

### 3.7 The Cholesterol‐Synthesis Pathway Genes Were Upregulated in Tia1^−/−^ Astrocytes Through Decreasing the Sequestration of Their mRNA in SGs

**FIGURE 8:**
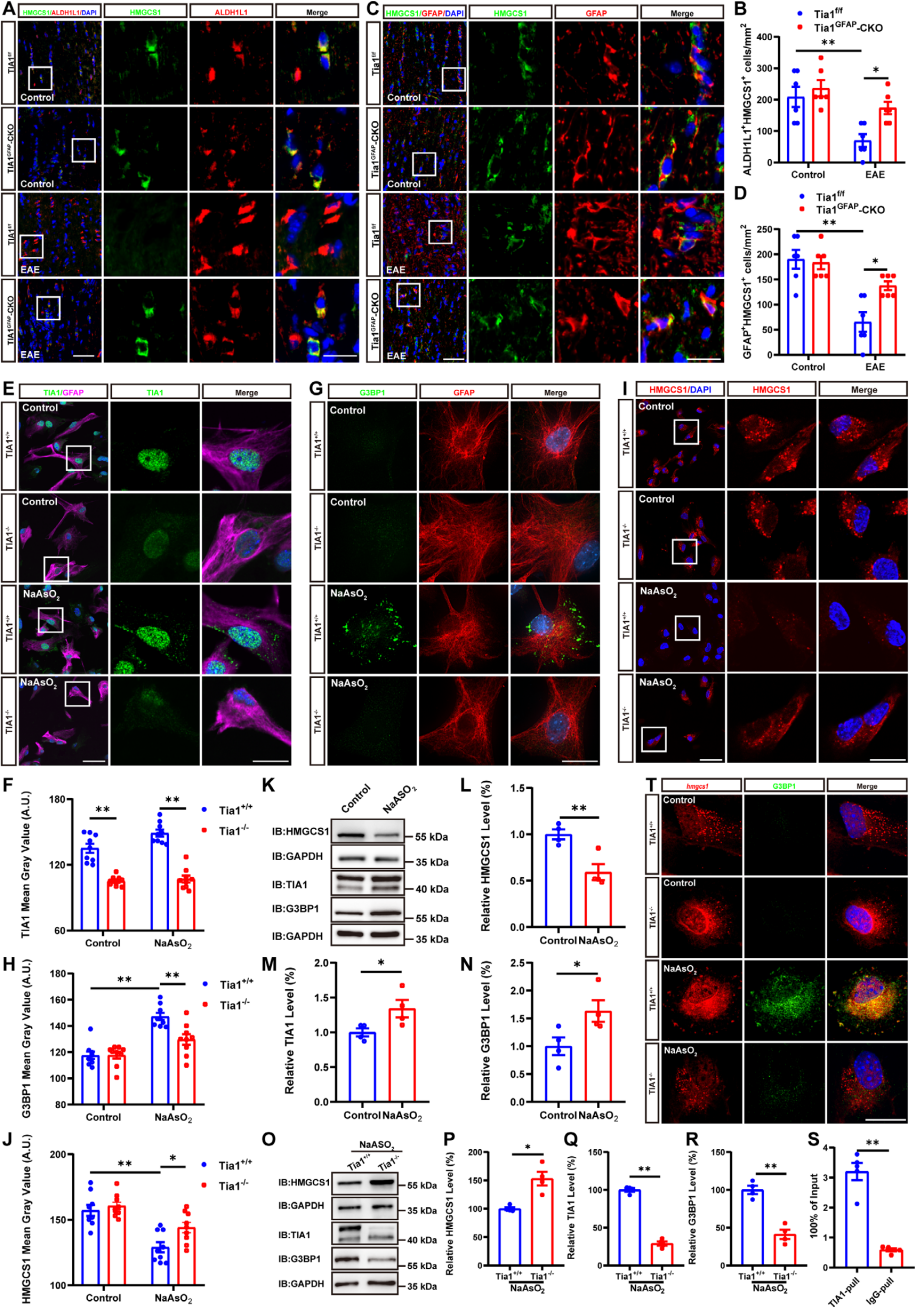
The cholesterol‐synthesis pathway genes were upregulated in *Tia1^−/−^* astrocytes through decreasing the sequestration of mRNA in SGs. (A) Double immunostaining of HMGCS1 (green) and ALDH1L1 (red) in the optic nerves of *Tia1* ^f/f^ and *Tia1* ^GFAP^‐CKO mice, *Tia1* ^f/f^ EAE and *Tia1* ^GFAP^‐CKO EAE mice. (B) Quantitative analysis of the density of ALDH1L1^+^/HMGCS1^+^ cells as shown in (A) (n = 6). (C) Double immunostaining of HMGCS1 (green) and GFAP (red) in the optic nerves of *Tia1* ^f/f^ and *Tia1* ^GFAP^‐CKO mice, *Tia1* ^f/f^ EAE and *Tia1* ^GFAP^‐CKO EAE mice. (D) Quantitative analysis of density of GFAP^+^/HMGCS1^+^ cells as shown in (C) (n = 6). (E, G, I) Double immunostaining of TIA1(green) and GFAP (far‐red) (E), G3BP1 (green) and GFAP (red) (G), and HMGCS1 (red) (I) in *Tia1* ^+/+^ astrocytes and *Tia1* ^−/−^ astrocytes, NaAsO_2_‐treated *Tia1* ^+/+^ astrocytes and NaAsO_2_‐treated *Tia1* ^−/−^ astrocytes. (F, H, J) Quantitative analysis of the mean intensity of TIA1 (F), G3BP1 (H) and HMGCS1 (J) as shown in (E) or (G) or (I), respectively (n = 9). (K) Western blot detected the HMGCS1, TIA1, and G3BP1 expression in the control and NaAsO_2_‐stimulated astrocytes. (L–N) Quantitative analysis of the HMGCS1 (L), TIA1 (M), and G3BP1 (N) protein levels as shown in (K) (n = 4, normalized to GAPDH). (O) Western blot detected the HMGCS1, TIA1 and G3BP1 expression in NaAsO_2_‐treated *Tia1* ^+/+^ astrocytes and NaAsO_2_‐treated *Tia1* ^−/−^ astrocytes. (P‐R) Quantitative analysis of the HMGCS1 (P), TIA1 (Q) and G3BP1 (R) protein levels as shown in (O) (n = 4, normalized to GAPDH). (S) Quantitative analysis of qRT‐PCR results from RIP assay to detect the interaction of TIA1 and *hmgcs1* mRNA in the optic nerves of *Tia1* ^f/f^ EAE mice (n = 5, normalized to the input RNA). (T) Typical images of G3BP1 (green) and *hmgcs1* mRNA‐FISH (red) in *Tia1* ^+/+^ astrocytes and *Tia1* ^−/−^ astrocytes, NaAsO_2_‐treated *Tia1* ^+/+^ astrocytes and NaAsO_2_‐treated *Tia1* ^−/−^ astrocytes. Scale bars, 50 and 20 µm (enlarge). ^*^p < 0.05, ^**^p < 0.01.

Consistent with the above results, interestingly, in *Tia1* ^GFAP^‐CKO EAE mice, double immunostaining of HMGCS1/ALDH1L1, HMGCS1/GFAP showed the up‐regulation of HMGCS1 in optic nerve astrocytes (Figure 8A–D). Moreover, Filipin staining showed that the free‐cholesterol signal was higher in optic nerves of *Tia1* ^GFAP^‐CKO EAE mice, compared with that in *Tia1* ^f/f^ EAE mice (Figure S6A,B). Consistently, TC levels in the optic nerves were higher in *Tia1* ^GFAP^‐CKO EAE mice than in *Tia1* ^f/f^ EAE mice (Figure S6C). To further confirm the results obtained in EAE mice, a stress model in cultured astrocytes was established in vitro. In this model, astrocytes were incubated with 0.5 mM sodium arsenite (NaAsO_2_) for 45 min prior to fixation. NaAsO_2_ exposure increased TIA1 and G3BP1 expression while decreasing HMGCS1 expression (Figure 8K–N). Meanwhile, SG formation was significantly decreased in *Tia1* ^−/−^ astrocytes (Figure 8E–H,O, Q,R). Interestingly, in this model, we found that HMGCS1 was decreased in *Tia1^+/+^* astrocytes; however, *Tia1* knockout suppressed the reduction of HMGCS1 in *Tia1^−/−^* astrocytes (Figure 8O,P). Immunostaining further confirmed a notable reduction in the fluorescence intensity of HMGCS1 in NaAsO_2_‐treated *Tia1^+/+^* astrocytes. However, compared with NaAsO_2_‐treated *Tia1^+/+^* astrocytes, the reduction of HMGCS1 was significantly inhibited in NaAsO_2_‐treated *Tia1^−/−^* astrocytes (Figure 8I,J).

We next examined whether the cholesterol‐synthesis pathway genes, such as *hmgcs1* mRNA, might be selectively sequestered into TIA1‐mediated SGs in astrocytes. Interestingly, as expected, RIP and qRT‐PCR experiments further confirmed the binding of TIA1 protein to *hmgcs1* mRNA in optic nerves of EAE mice (Figure 8S). Furthermore, FISH assays further showed partial co‐localization of *hmgcs1* mRNA and SGs marker G3BP1 in NaAsO_2_‐treated *Tia1^+/+^* astrocytes, and this co‐localization was significantly reduced in NaAsO_2_‐treated *Tia1^−/−^* astrocytes (Figure 8T). Taken together, these results suggested the cholesterol‐synthesis pathway protein HMGCS1 was upregulated in *Tia1^−/−^* astrocytes through decreasing the sequestration of their mRNA in SGs, which indicated that TIA1‐mediated SGs contributed to the demyelination of the optic nerves through sequestration of the cholesterol‐synthesis pathway genes' mRNA into SGs in astrocytes.

### 3.8 Upregulation of Cholesterol‐Synthesis Pathway Promoted the Expression of HMGCS1 and Partially Restored the Demyelination Deficits in Tia1^GFAP^‐CKO EAE Mice

**FIGURE 9:**
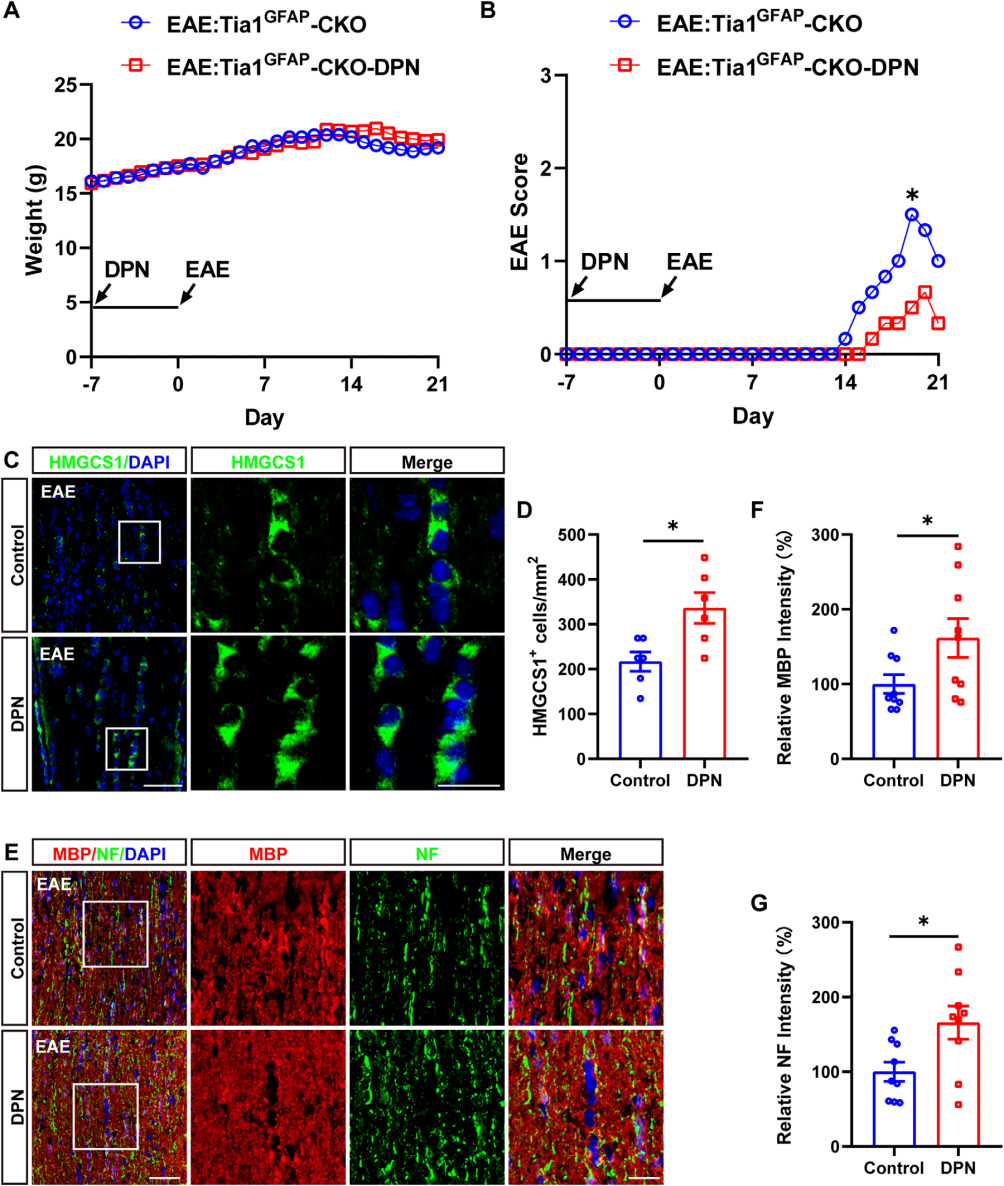
Upregulating cholesterol‐synthesis pathways partially rescued the demyelination deficits in *Tia1* ^GFAP^‐CKO EAE mice. (A,B) The body weight (A) and EAE score (B) of *Tia1* ^GFAP^‐CKO mice treated with vehicle or DPN from 0 to 21 dpi during the EAE modeling process (n = 3). (C) Immunostaining of HMGCS1 (green) in the optic nerves of *Tia1* ^GFAP^‐CKO mice treated with vehicle or DPN. (D) Quantitative analysis of the density of HMGCS1^+^ cells as shown in (C) (n = 6). (E) Double immunostaining of MBP (red) and NF (green) in the optic nerves of *Tia1* ^GFAP^‐CKO mice treated with vehicle or DPN. (F,G) Quantitative analysis of the relative MBP intensity (F) or NF intensity (G) as shown in (E) (n = 6, normalized to *Tia1* ^GFAP^‐CKO mice treated with vehicle). Scale bars, 50 and 20 µm (enlarge). ^*^p < 0.05, ^**^p < 0.01.

We next examined whether upregulation of the cholesterol‐synthesis pathway further alleviated the demyelination deficits in *Tia1* ^GFAP^‐CKO EAE mice. Previous studies have shown that administering ERβ ligands, diarylpropionitrile (DPN), during remyelination increased the expression of genes linked to cholesterol synthesis [25], then inhibited demyelination deficits in EAE mice [34]. Then, DPN was applied to *Tia1* ^GFAP^‐CKO EAE mice to mitigate demyelination deficits through upregulating HMGCS1 expression in *Tia1* ^GFAP^‐CKO EAE mice. As expected, we found that DPN treatment improved the functional recovery in *Tia1* ^GFAP^‐CKO EAE mice, compared with that in vehicle‐treated *Tia1* ^GFAP^‐CKO EAE mice (Figure 9A,B). Furthermore, immunostaining revealed that DPN treatment partially restored the decrease of HMGCS1, MBP, and NF expression in the optic nerves of *Tia1* ^GFAP^‐CKO EAE mice (Figure 9C–G), compared with that in vehicle‐treated *Tia1* ^GFAP^‐CKO EAE mice. Taken together, these results suggested that upregulation of HMGCS1 partially restored the demyelination deficits in *Tia1* ^GFAP^‐CKO EAE mice.

## 4 Discussion

**FIGURE 10:**
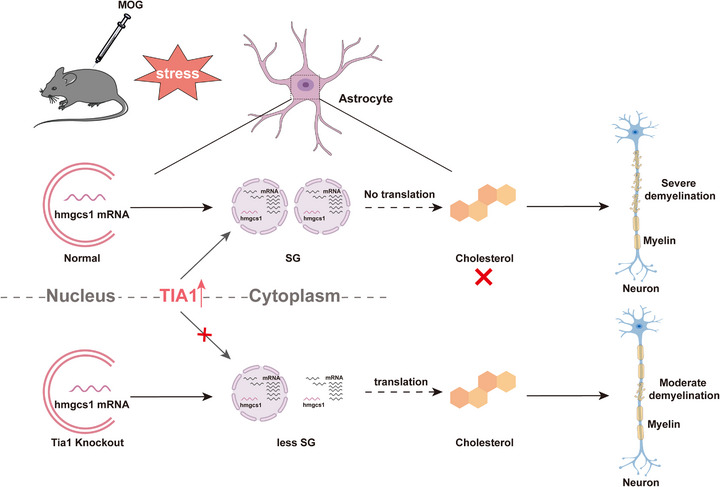
Working model. Astrocytic TIA1‐mediated SGs dynamics in promoting demyelination through sequestering mRNA of cholesterol synthesis genes. In this model, astrocytic TIA1‐mediated SGs were increased in optic nerves of EAE mice, leading to the downregulation of cholesterol synthesis genes such as HMGCS1 through sequestration of their mRNA into SGs, which exacerbated the demyelination of the optic nerves in EAE mice.

In our study, we provide evidence for the roles and mechanisms of TIA1‐mediated SGs in EAE‐ON and propose a working model (Figure 10). In this model, astrocytic TIA1‐mediated SGs were increased in the optic nerves of EAE mice, leading to the downregulation of cholesterol synthesis genes such as *HMGCS1* through sequestration of their mRNA into SGs, which exacerbated the demyelination of the optic nerves in EAE mice. Our findings reveal an unrecognized function of TIA1‐mediated SGs in MS‐ON, and identify that astrocytic TIA1‐mediated SGs promote the demyelination of ON through sequestering mRNA of cholesterol synthesis genes.

Molecular analysis indicates an increased expression of SG‐related genes in MS patients, and immunoreactive large SGs formation in neurons from MS brains, suggesting a potential basis for neuronal damage in MS [13]. Neurons in the MS brain showed the formation of large SGs immunoreactive for TIA1, as well as decreased or complete lack of nuclear TIA1 staining [14]. Research has shown that in EAE models, the formation of SGs in neurons, astrocytes, oligodendrocytes, and microglia may be increased within and around demyelinated plaques [35]. Consistent with these previous studies, our study also showed that SGs and TIA1 were increased in retinal neurons and optic nerve astrocytes of EAE mice (Figure 1). Furthermore, in an in vitro model of NaAsO_2_‐stimulated astrocytes, we found that SG formation was substantially reduced by *Tia1* depletion (Figure 8E–H,O,Q,R). These results indicate that TIA1‐mediated SGs play critical roles in MS patients or EAE‐ON mice.

Under pathologic conditions, SGs may persist and have been shown to lead to decreased cell survival and the induction of proapoptotic pathways [36, 37, 38]. Augmented SGs formation has also been shown to decrease cell viability [37]. Salapa and colleagues have previously provided evidence of SG dysfunction that may underlie neuronal damage in MS disorder [14]. Consistent with these studies, in our study, *Tia1* knockout in the CNS did not affect the development of mice, but suppressed the demyelination and inflammatory infiltration in optic nerves, and reduced the loss of RGCs and the inflammatory infiltration in retina after EAE induction (Figures 2, 3, 4), suggesting that TIA1‐mediated SGs promoted EAE‐ON.

Demyelination is a primary pathological characteristic of MS [39]. Cholesterol synthesis metabolism within the central nervous system play a critical role in both axonal demyelination and remyelination. Although demyelination is a consequence of autoimmune attacks in EAE, limited remyelination may be partially attributed to decreased cholesterol synthesis within astrocytes [32]. Cholesterol constitutes a major component of myelin sheaths, yet the blood‐brain barrier largely impedes the entry of peripheral cholesterol into the brain [40]. It has been reported that neurons have a lower ability to compensate for cholesterol deficits by *de novo* synthesis than astrocytes [41]. During adulthood, neurons largely discontinue cholesterol synthesis, relying predominantly on astrocytes for their cholesterol supply [42]. Hence, in adulthood, the principal synthesis of cholesterol occurs within astrocytes in the brain, which is then transported to both oligodendrocytes and neurons [40]. In addition, previous studies have shown that astrocytic YAP is able to prevent spinal cord demyelination by promoting the expression of *HMGCS1*, a cholesterol synthesis gene [34].

Consistent with these previous studies, in this study, interestingly, mRNA sequencing revealed that cholesterol synthesis genes such as *HMGCS1* were down‐regulated in the optic nerves of *Tia1* ^f/f^ EAE mice and up‐regulated in the optic nerves of *Tia1* ^Nestin^‐CKO EAE mice (Figure 5A–C). Recent studies have reported that cholesterol synthesis genes, such as *HMGCS1* expression is reduced in astrocytes of the optic nerves in EAE models [32], which is consistent with our study. Research also indicates that G3BP1‐dependent SGs limit axonal mRNA translation and nerve regeneration [43]. Under in vitro stress conditions, mRNA related to myelin is sequestered within SGs within oligodendrocytes [44]. Prolonged sequestration of myelin‐related mRNA within SGs may have adverse effects on the remyelination process [45]. However, it remains unclear whether these cholesterol synthesis genes within astrocytes are regulated via SGs during the EAE process. We suspect that *hmgcs1* mRNA is translational blocked after selective sequestration into SGs, resulting in decreased cholesterol expression and worsening demyelination in optic neuritis of EAE mice.

To further test the above conjecture, the NaAsO_2_‐induced SGs model was established in astrocytes. Simultaneously, as an RNA‐binding protein, the RIP‐qPCR experiments showed that TIA1 directly bound with hmgcs1 mRNA, suggesting a potential role in modulating its translation (Figure 8S). Consistently, it has been reported that SG‐proximity transcriptomic analyses using G3BP1‐miniSOG CAP‐seq have identified *HMGCS1*, together with other cholesterol‐synthesis pathway genes such as *HMGCR* and *SQLE*, within the SG‐proximal RNA set in NaAsO_2_‐treated U‐2 OS cells, indicating the spatial association of these mRNAs with canonical stress granule components [46]. Unfortunately, the mechanism of SGs breakdown and degradation is still unclear, but it is well worth investigating in our future studies. In MS clinical trials, statin treatment has primarily targeted anti‐inflammatory effects. However, the question of whether statins can penetrate the CNS during disease progression to influence cholesterol homeostasis in astrocytes in either a detrimental or beneficial manner remains a topic requiring thorough investigation [47, 48, 49]. Collectively, data from the spinal cord and optic nerves suggest that targeting cholesterol synthesis in astrocytes in MS may affect walking and vision, respectively [34]. As reported previously, as a response to short‐term stress, SGs are neuroprotective initially, while they are pernicious upon the chronic stress. NaAsO_2_‐stimulated astrocytes are a short‐term stress model in vitro, so there is a limitation that the in vitro model we used is not very compatible with the EAE model in vivo, which induces chronic stress in mice. This issue warrants seeking an in vitro model that is more consistent with the EAE model in future studies.

In this study, TIA1 was upregulated in retinal neurons and optic nerve astrocytes of EAE mice, and we used *Tia1* ^Nestin^‐CKO mice to explore the role of TIA1‐mediated SGs in MS‐ON. According to the sequencing results, we further used *Tia1* ^GFAP^‐CKO mice to explore the role of astrocytic TIA1 in MS‐ON. However, the EAE score of *Tia1* ^Nestin^‐CKO mice was lower than that of *Tia1* ^GFAP^‐CKO mice; we couldn't entirely exclude the role of TIA1 in other cells, such as neurons and oligodendrocytes. It should also be noted that, owing to bulk‐RNA sequencing, we cannot entirely exclude a contribution from oligodendrocytes or other cell types to the observed up‐regulation of cholesterol‐metabolism genes, and therefore cannot establish whether this change is astrocyte‐specific. Consistently, although DPN treatment increased overall HMGCS1 expression in the optic nerves, the cell‐type specificity of this effect was not determined and may involve contributions from multiple cell populations. Moreover, while GFAP is always regarded as a marker for astrocytes, indeed, it is also expressed in some neural stem cells. Therefore, more specific astrocytic Cre mice would be used to test the detailed role and mechanism of TIA1 in astrocytic SGs in the future.

In conclusion, our study identified an unknown role of TIA1‐mediated SGs in aggravating the demyelination optic nerves in EAE mice by regulating cholesterol synthesis genes. In the future, reducing the formation of SGs or increasing the expression of HMGCS1 by drugs may have synergistic effects to promote remyelination and treat demyelinating diseases such as MS‐ON.

## Conflicts of Interest

The authors declare no conflicts of interest.

## Supporting information




**Supporting File**: advs74397‐sup‐0001‐SuppMat.docx.

## Data Availability

The data that support the findings of this study are available in the supplementary material of this article.
