# Supplementary material for: Astrocytic TIA1‐Mediated Stress Granules Promote the Demyelination of Optic Neuritis by Sequestering mRNA of Cholesterol Synthesis Genes in an Experimental Autoimmune Encephalomyelitis Model
Source: Adv Sci (Weinh). 2026 Feb 27;13(23):e20299. doi: 10.1002/advs.202520299 (PMC13104089; doi:10.1002/advs.202520299)
Supplement: Supplementary file 1 — Supporting File: advs74397‐sup‐0001‐SuppMat.docx. [file ADVS-13-e20299-s001.docx]

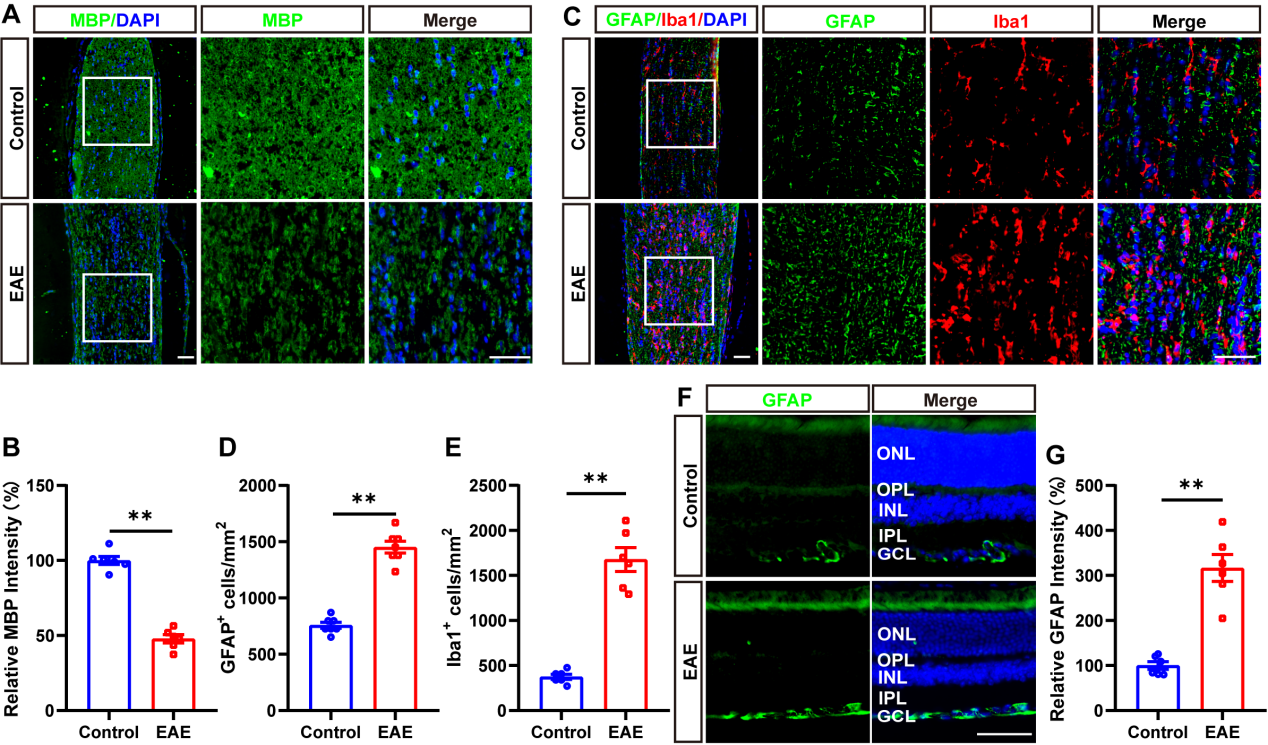


**Figure. S1 Establishment of EAE-ON mice model.**

(**A**) Immunostaining of MBP (green) in the optic nerves of control and EAE mice. (**B**) Quantitative analysis of the relative MBP intensity as shown in (**A**) (n = 6, normalized to control). (**C**) Double immunostaining of GFAP (green) and Iba1 (red) in the optic nerves of control and EAE mice. (**D-E**) Quantitative analysis of the density of GFAP^+^ cells (**D**) or Iba1^+^ cells (**E**) as shown in (**C**) (n = 6). (**F**) Immunostaining of GFAP (green) in the retina of control and EAE mice. (**G**) Quantitative analysis of the relative GFAP intensity as shown in (**F**) (n = 6, normalized to control). Scale bars, 50 μm and 20 μm (enlarge). *^**^P < 0.01*.


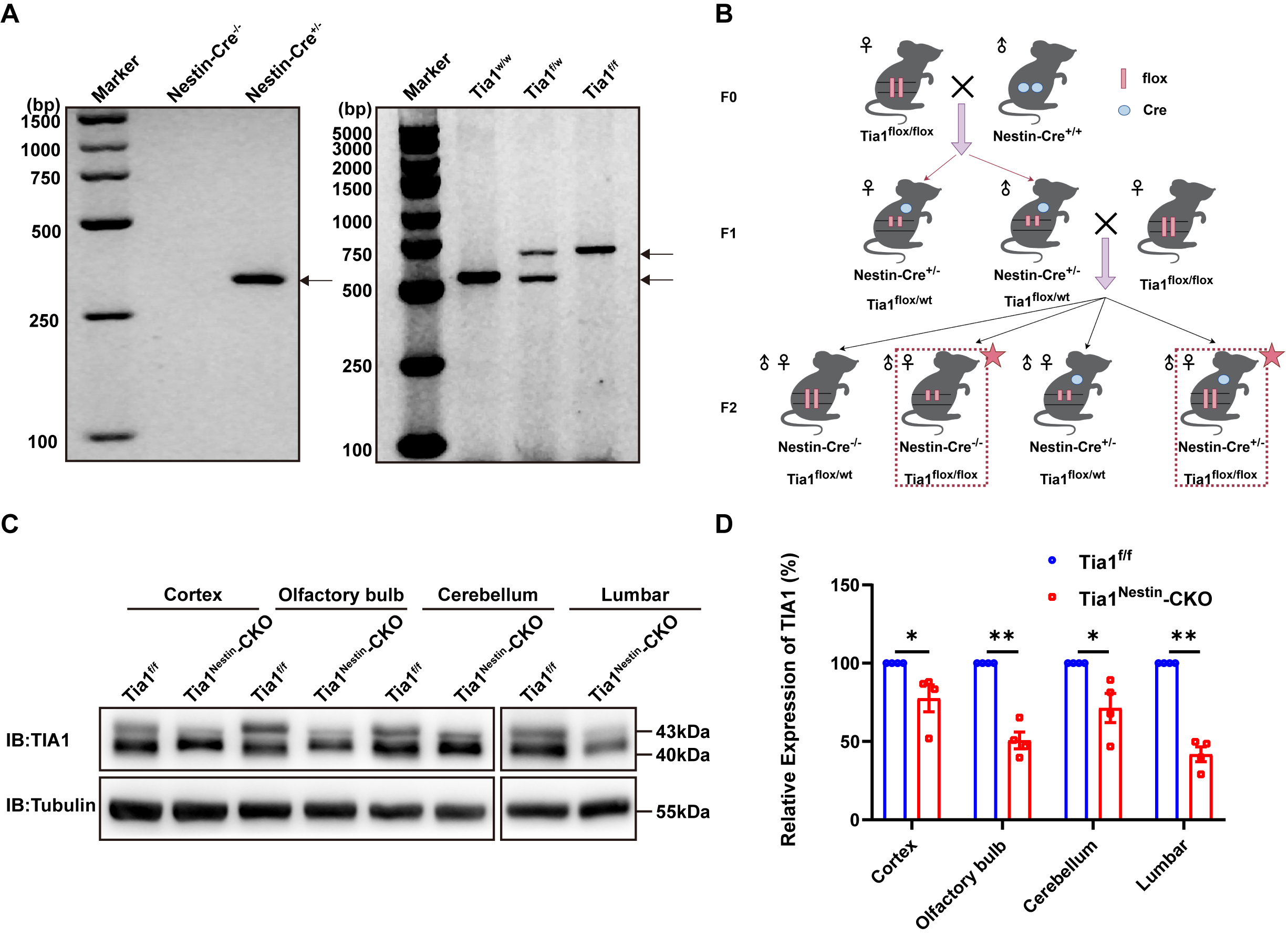


**Figure. S2 Identification of *Tia1*^Nestin^-CKO mice.** (**A**) Agarose gel electrophoresis identification of *Tia1*^Nestin^-CKO mice. (**B**) Schematic flow chart illustrating the breeding strategy used to generate *Tia1* conditional knockout mice in neural stem cells and their progeny (Nestin-Cre; *Tia1*^flox/flox^). (**C**) Western blot analysis of TIA1 expression in the cortex, olfactory bulb, cerebellum and spinal cord from 6-week-old female *Tia1*^f/f^ mice and *Tia1*^Nestin^-CKO mice. (**D**) Quantitative analysis of the relative TIA1 levels as shown in (**C**) (n = 4, normalized to β-tubulin). *^*^P < 0.05, ^**^P < 0.01*.

**Figure. S3 The normal development of optic nerves and retina in *Tia1*^Nestin^-CKO mice.**

(**A**) HE staining of optic nerves of *Tia1*^f/f^ and *Tia1*^Nestin^-CKO mice. (**B**) Quantitative analysis of the density of inflammatory cells as shown in (**A**) (n = 6). (**C, E**) Immunostaining of GFAP (red) (**C**) or Iba1 (red) (**E**) in the optic nerves of *Tia1*^f/f^ and *Tia1*^Nestin^-CKO mice. (**D, F**) Quantitative analysis of the density of GFAP^+^ cells (**D**) or the density of Iba1^+^ cells (**F**) as shown in (**C**), or (**E**), respectively (n = 6). (**G**, **I, K, M,**) Immunostaining of RBPMS (green) (**G**), or NeuN (green) (**I**), or GFAP (green) (**K**), or Iba1 (red) (**M**) in the retina of *Tia1*^f/f^ and *Tia1*^Nestin^-CKO mice. (**H**, **J, L, N**) Quantitative analysis of the density of RBPMS^+^ cells (**H**), the density of NeuN^+^ cells (**J**), the relative GFAP intensity (**L**), the density of Iba1^+^ cells (**N**) as shown in (**G**), or (**I**), or (**K**), or (**M**), respectively (n = 6). Scale bars, 20 μm. n.s., not significant.


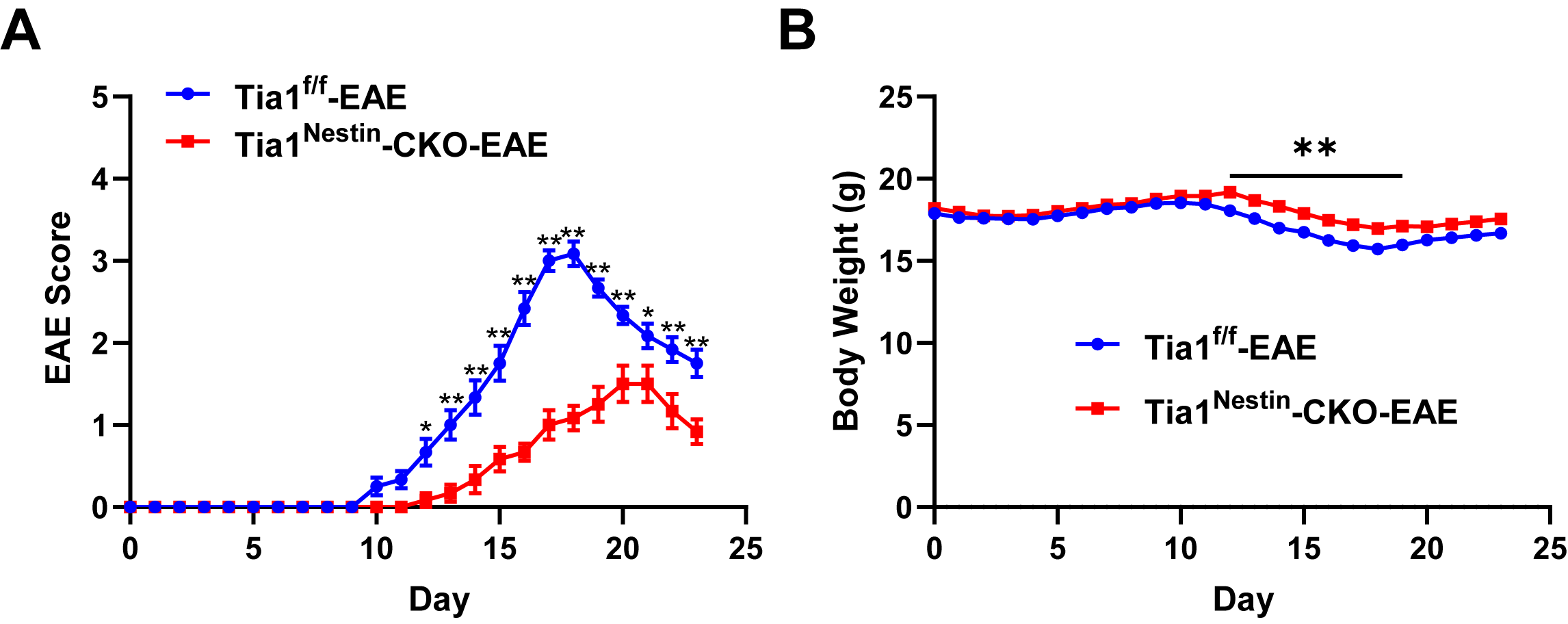


**Figure. S4 Extended clinical scores and body-weight curves during EAE induction.** (**A**) The EAE score of *Tia1*^f/f^ and *Tia1*^Nestin^-CKO mice 0 to 23 dpi during the process of EAE modeling (n = 6). (**B**) The body weight of *Tia1*^f/f^ and *Tia1*^Nestin^-CKO mice from 0 to 23 dpi during the process of EAE modeling (n = 6).


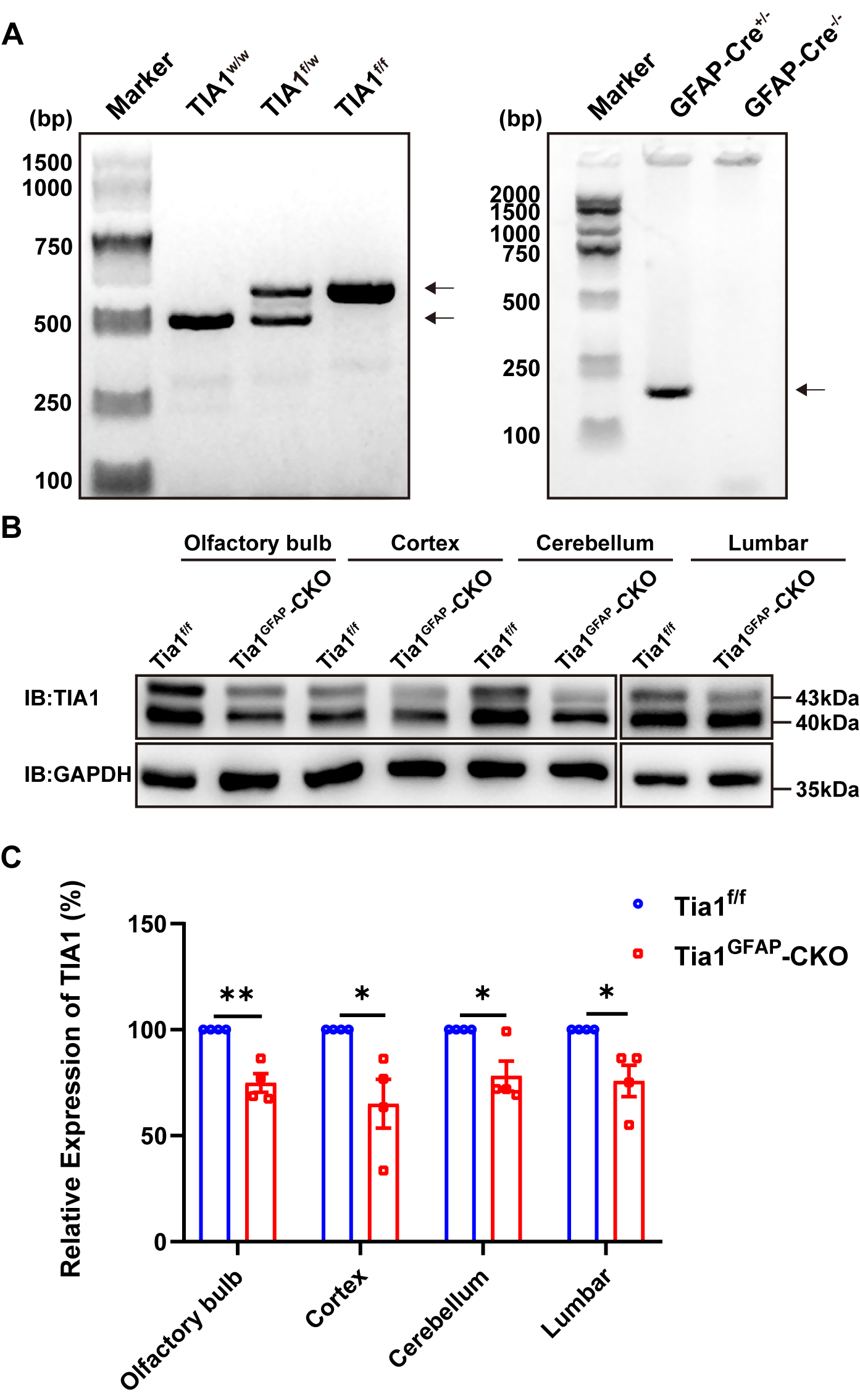


**Figure. S5 Identification of *Tia1*^GFAP^-CKO mice.** (**A**) Agarose gel electrophoresis identification of *Tia1*^GFAP^-CKO mice. (**B**) Western blot analysis of TIA1 expression in the olfactory bulb, cortex, cerebellum and spinal cord of 6-week-old female *Tia1*^f/f^ mice and *Tia1*^GFAP^-CKO mice. (**C**) Quantitative analysis of the relative TIA1 levels as shown in (**B**) (n = 4, normalized to GAPDH). *^*^P < 0.05, ^**^P < 0.01*.


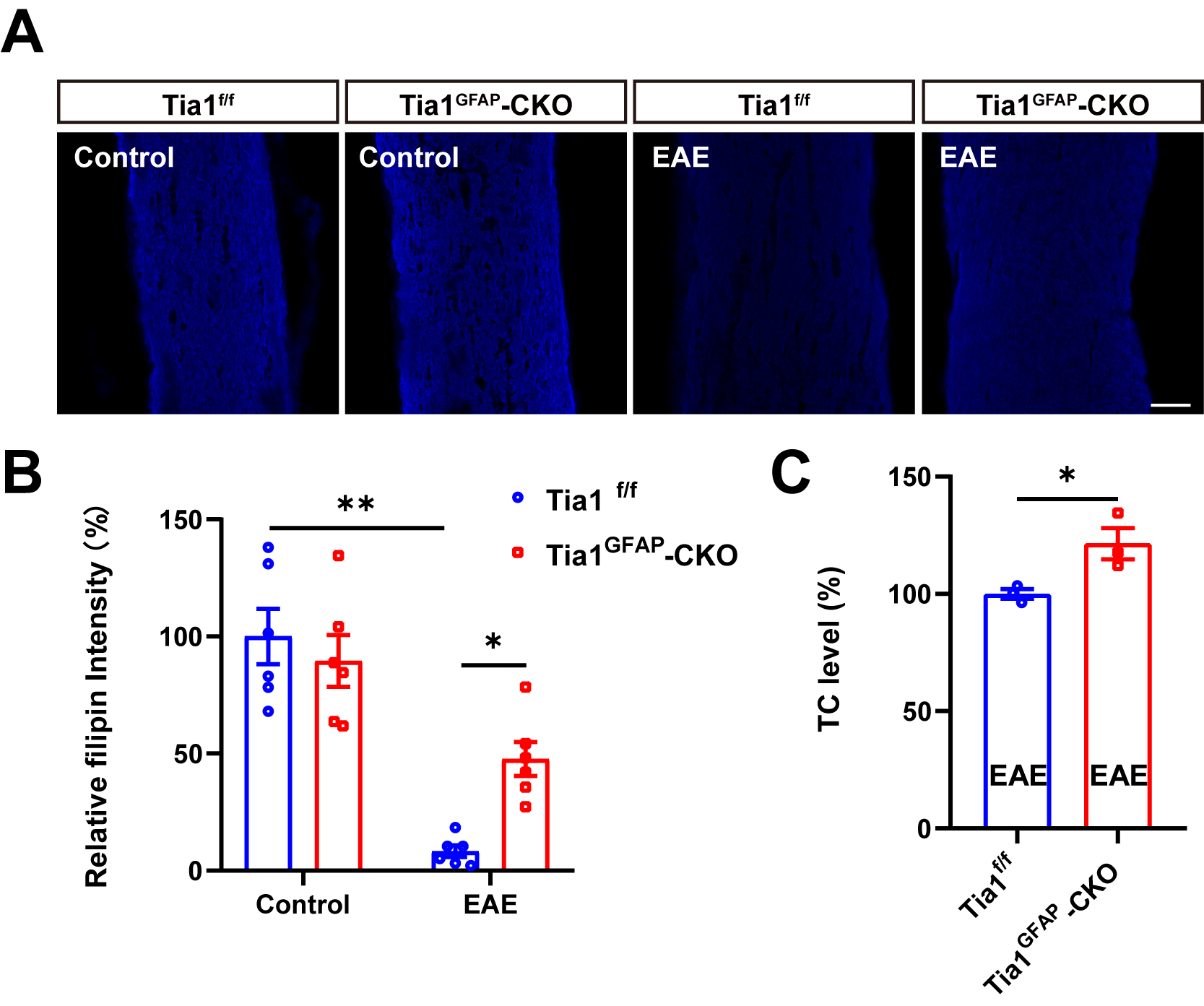


**Figure. S6 Cholesterol analysis in optic nerves.** (**A**) Filipin staining in the optic nerves of *Tia1*^f/f^ and *Tia1*^GFAP^-CKO mice, *Tia1*^f/f^ EAE and *Tia1*^GFAP^-CKO EAE mice. (**B**) Quantitative analysis of the relative filipin intensity as shown in (A) (n = 6, normalized to *Tia1*^f/f^ mice). (**C**) TC level in the optic nerves of *Tia1*^f/f^ EAE and *Tia1*^GFAP^-CKO EAE mice (n = 3, normalized to *Tia1*^f/f^ EAE mice). Scale bars, 50 μm. *^*^P < 0.05, ^**^P < 0.01*.
